# Synergistic Dual Targeting of Thioredoxin and Glutathione Systems Irrespective of p53 in Glioblastoma Stem Cells

**DOI:** 10.3390/antiox13101201

**Published:** 2024-10-03

**Authors:** Fatemeh Jamali, Katherine Lan, Paul Daniel, Kevin Petrecca, Siham Sabri, Bassam Abdulkarim

**Affiliations:** 1 Pathology Graduate and Postdoctoral Studies Program, Department of Pathology, McGill University, Montreal, QC H3A 2B4, Canada; fatemeh.jamali@mail.mcgill.ca; 2 Cancer Research Program, Research Institute of the McGill University Health Centre, 1001 Decarie Blvd, Montreal, QC H4A 3J1, Canada; katherine.lan@mail.mcgill.ca; 3 Division of Experimental Medicine, McGill University, Montreal, QC H4A 3J1, Canada; 4 Centre for Cancer Research, Department of Molecular and Translational Science, Hudson Institute of Medical Research, Faculty of Medicine, Monash University, Clayton, VIC 3168, Australia; paul.daniel@hudson.org.au; 5 Department of Neurology and Neurosurgery, Faculty of Medicine, McGill University, Montreal, QC H3A 1A1, Canada; kevin.petrecca@mcgill.ca; 6 Department of Oncology, Division of Radiation Oncology, McGill University, Montreal, QC H4A 3T2, Canada

**Keywords:** Glioblastoma, Glioblastoma stem cells (GSCs), auranofin, thioredoxin reductase, oxidative stress, glutathione (GSH), reactive oxygen species (ROS), piperlongumine, antioxidant, drug repurposing

## Abstract

Glioblastoma (GBM) is an incurable primary brain cancer characterized by increased reactive oxygen species (ROS) production. The redox-sensitive tumor suppressor gene *TP53*, wild-type (wt) for 70% of patients, regulates redox homeostasis. Glioblastoma stem cells (GSCs) increase thioredoxin (Trx) and glutathione (GSH) antioxidant systems as survival redox-adaptive mechanisms to maintain ROS below the cytotoxic threshold. Auranofin, an FDA-approved anti-rheumatoid drug, inhibits thioredoxin reductase 1 (TrxR1). L-buthionine sulfoximine (L-BSO) and the natural product piperlongumine (PPL) inhibit the GSH system. We evaluated the cytotoxic effects of Auranofin alone and in combination with L-BSO or PPL in GBM cell lines and GSCs with a known *TP53* status. The Cancer Genome Atlas/GBM analysis revealed a significant positive correlation between wtp53 and TrxR1 expression in GBM. Auranofin induced ROS-dependent cytotoxicity within a micromolar range in GSCs. Auranofin decreased TrxR1 expression, AKT (Ser-473) phosphorylation, and increased p53, p21, and PARP-1 apoptotic cleavage in wtp53-GSCs, while mutant-p53 was decreased in a mutant-p53 GSC line. Additionally, p53-knockdown in a wtp53-GSC line decreased TrxR1 expression and significantly increased sensitivity to Auranofin, suggesting the role of wtp53 as a negative redox-sensitive mechanism in response to Auranofin in GSCs. The combination of Auranofin and L-BSO synergistically increased ROS, decreased IC50s, and induced long-term cytotoxicity irrespective of p53 in GBM cell lines and GSCs. Intriguingly, Auranofin increased the expression of glutathione S-transferase pi-1 (GSTP-1), a target of PPL. Combining Auranofin with PPL synergistically decreased IC50s to a nanomolar range in GSCs, supporting the potential to repurpose Auranofin and PPL in GBM.

## 1. Introduction

Glioblastoma (GBM) is classified as isocitrate dehydrogenase-wildtype, grade 4, adult-type diffuse gliomas, according to the 2021 World Health Organization classification of tumors of the central nervous system (CNS) [1]. Despite a multimodal standard-of-care involving surgery, radiation therapy (RT) and temozolomide (TMZ) chemotherapy, the median survival is only 12–15 months for patients newly diagnosed with GBM [2]. GBM, the most common primary malignant CNS tumor (50.9%), remains the most lethal brain cancer, with a five-year relative survival rate of 5.6% for patients over 40 years old [3]. Many biological and molecular challenges contribute to its therapeutic resistance and inevitable tumor recurrence, hampering further advances in GBM treatment [4]. Glioblastoma stem cells (GSCs), a small subpopulation of GBM cells, drive tumor initiation and play a key role in GBM’s resistance to chemoradiation and tumor recurrence [5,6,7]. RT and TMZ induce DNA damage and generate high levels of reactive oxygen species (ROS). However, GSCs adapt the main endogenous antioxidant systems, thioredoxin (Trx) and glutathione (GSH) to reduce ROS, maintaining a reduced milieu and promoting their survival [5,8,9,10]. ROS are derived from molecular oxygen upon its incomplete reduction during reduction-oxidation (redox) reactions that produce cellular energy for aerobic organisms [11]. Due to high basal metabolic activity in the brain, and its vulnerability to ROS-induced oxidative damage, the regulation of the Trx and GSH systems is crucial for maintaining physiological levels of ROS [10].

The Trx system comprised of NADPH and ubiquitous proteins. Trx and thioredoxin reductase (TrxR) protects cells from the deleterious effects of ROS generated during cellular mitochondrial respiration [12]. Mammalian TrxRs are homodimeric flavoproteins with a FAD prosthetic group, NADPH binding site, redox-active disulfide, and selenocysteine residues essential to their catalytic activity. TrxR, a key antioxidant enzyme, takes electrons from NADPH to reduce the disulfide in its substrate Trx [13]. This catalytic reaction maintains Trx in a reduced state [Trx-(SH)_2_]. The reduced form of Trx transfers reducing equivalents to oxidized disulfides within target molecules, leading to their reduction. Reduced Trx interacts with a large array of important downstream redox-sensitive signaling molecules and transcription factors [10,14]. TrxR overexpression in several cancers, including GBM, has been correlated with increased malignancy grade, tumor recurrence, and drug resistance, rendering it an attractive target for cancer therapy [9,10].

Trx and GSH control the cellular redox environment to keep redox homeostasis in physiological conditions. GSH, a major antioxidant system that includes GSH, a non-enzymatic antioxidant tripeptide (γ-L-glutamyl-L-cysteinyl-glycine), and GSH-metabolizing enzymes, also uses NADPH as a source of reducing equivalents. In cancer cells, GSH acts as an antioxidant ROS scavenger and signaling molecule for several oncogenes [15]. GSH-metabolizing and synthesizing enzymes, such as gamma-glutamate-cysteine ligase catalytic (GCLC), glutathione peroxidases (GPXs), and glutathione transferases (GSTs), regulate GSH levels for ROS detoxification in the brain [10,16]. Increased intracellular GSH levels and overexpression of GSH-metabolizing enzymes, such as glutathione transferase pi-1 (GSTP-1), have been correlated with drug resistance and reported in TMZ-resistant GBM cells [16,17]. 

ROS acts as a double-edged sword, promoting cell growth and contributing to cell signaling at low levels while inducing cell death at high levels [11,18]. Increased cell proliferation and metabolism generate higher ROS levels in cancer cells compared to normal cells [18]. Anti-cancer ROS-inducers, including RT and TMZ, exploit the intrinsically high ROS levels in cancer cells as a therapeutic vulnerability to generate ROS and selectively kill cancer cells [19,20]. However, the Trx and GSH antioxidant systems protect these cells from ROS-induced damage, keeping ROS levels below the threshold of oxidative damage to maintain their redox homeostasis [9,10,15]. Increasing evidence supports the role of important redox-sensitive oncogenes and tumor suppressor genes in ROS regulation within GBM [9,21,22]. For instance, the tumor suppressor protein p53 converts various redox signals to select specific p53-target genes that affect ROS levels and determine cell fate [21]. The crosstalk between p53 and the Trx and GSH antioxidant systems contributes to the regulation of these redox processes [23]. Wild-type (wt) *TP53* acts as a redox-sensitive transcription factor involved in cell cycle arrest, apoptosis, DNA repair, and fine-tuned regulation of either antioxidant or pro-oxidant transcriptional targets depending on basal cellular p53 function in a reducing environment [22]. Previous studies reported the susceptibility of wtp53 to oxidation, suggesting the requirement for a reducing environment for the proper folding of wtp53 to restore DNA binding in vitro [24]. Oxidative post-translational changes of the redox-sensitive cysteine residues in p53’s DNA-binding core domain affect p53’s conformational tetrameric structure, inhibiting DNA binding and its transcriptional activities [25]. Increased expression of TrxR was associated with increased DNA binding activity in wtp53 [26]. The overexpression of human wtp53 strikingly decreased TrxR mRNA and triggered the lethal accumulation of high ROS levels in yeast [27]. The *GSTP1* gene emerged as a direct downstream transcriptional target of wtp53 [28], suggesting another level of the crosstalk between wtp53 and both the Trx and GSH antioxidant systems.

Alterations in ROS signaling due to an imbalance in the Trx and GSH antioxidant systems in different cancers [9,29,30,31].provides the rationale to design and use a combination of specific pro-oxidative TrxR and GSH inhibitors to reach lethal ROS levels. Earlier studies reported compensation mechanisms that counteract inhibition of Trx or GSH systems, a culprit for the failure of strategies targeting only one antioxidant system [32]. TrxR1-deficient mice exhibit increased sensitivity to buthionine sulfoximine (BSO), a GSH synthesis inhibitor [33]. Following TrxR1 inhibition and the loss of its activity, the GSH system serves as a backup to maintain Trx1 reduced. Accordingly, a combination of TrxR1 and GSH inhibitors oxidized Trx1 and induced cell death in Hela cells [34]. While a combination of TrxR1 and GSH inhibitors has the potential to cutback the mutual compensation between antioxidant systems, there is a lack of strategies that effectively exploit the dual inhibition of these antioxidant pathways in GBM. Furthermore, thus far, the role of p53 in response to concomitant inhibition of the Trx and GSH antioxidant systems has not been investigated in GSCs.

In this study, we hypothesized that the combination of ROS-inducer agents to co-target the Trx and GSH antioxidant systems might synergistically increase ROS to a lethal threshold for both GBM cell lines and GSCs. We aimed to evaluate the cytotoxic, molecular, and ROS-inducing effects of Trx and GSH inhibitors, while taking into account *TP53* status in the GSCs. Specifically, we investigated the effects of repurposing Auranofin (Au), a known TrxR1 inhibitor, in combination with GSH-targeting agents such as Piperlongumine (PPL) and L-buthionine-sulfoximine (L-BSO). Au, a gold(I)-containing compound that the FDA approved for the treatment of rheumatoid arthritis in 1985, is no longer used as a first-line treatment due to the availability of a new class of disease-modifying antirheumatic drugs. Au interacts directly and irreversibly with selenocysteine residues at the active site of TrxR1, leading to inhibition of its antioxidant activity and a subsequent ROS increase [35]. L-BSO is a synthetic inhibitor of GSH synthesis that targets the rate-limiting enzyme γ-GCS and inhibits the uptake of cysteine, the GSH precursor [36]. PPL is a natural alkaloid found in the plant Piper longum Linn that directly inhibits GSTP-1 and exhibits anti-cancer properties [17,37]. As such, the primary objectives of our research were twofold: first, to evaluate the cytotoxic effects of combined inhibition of the Trx and GSH systems in both GBM cell lines and GSCs, and second, to elucidate the role of wtp53 in the sensitivity of GSCs to this dual-targeting strategy.

We leveraged the pro-oxidant effects of Au to increase ROS and induce cytotoxic effects in GSCs and GBM established cell lines through a ROS-dependent mechanism. In silico analysis of GBM patients’ datasets showed a significant positive correlation between wtp53 and TrxR1 expression. In accordance with these findings, the knockdown of wtp53 in a GSC line drastically decreased TrxR1 expression and increased its sensitivity to Au, suggesting the role of wtp53 as a negative redox-sensitive mechanism involved in response of GSCs to TrxR1 inhibition. Combining Au with L-BSO demonstrated synergistic short-term and long-term cytotoxicity irrespective of p53 status in both wtp53 (U87MG GBM cell line, OPK161, OPK49 and isogenic OPK49 shRNA knockdown for P53 GSCs) and mutp53 (T98G GBM cell line and OPK257 GSC). Furthermore, synergistic combination of Au with PPL decreased AU IC50 to a nanomolar range, corroborating the vulnerability of GSCs to dual targeting of the Trx and GSH systems. Collectively, these results highlight the significance of targeting both the Trx and GSH systems to bypass the antioxidant role of wtp53-TrxR1 axis, induce lethal levels of ROS in both GBM cell lines and GSCs and open new avenues for the potential to repurpose Au in combination with a GSH-targeting strategy in GBM.

## 2. Materials and Methods

### 2.1. GBM Cell Lines and GSC Culture, Treatment, and Transfection

The human established GBM cell lines U87MG and T98G, obtained from the American Type Culture Collection (Manassas, VA, USA) were maintained in Dulbecco’s Modified Eagle’s Medium (DMEM), supplemented with 10% fetal bovine serum (FBS) and 1× penicillin-streptomycin. Cells were passaged using 5-to-8-min incubation with EDTA 0.53 mM cell detachment solution (Wisent Inc., Saint-Jean-Baptiste, QC, Canada). The GSC lines OPK161, OPK257, and OPK49 isolated from patients newly diagnosed with GBM in the laboratory of Dr. K. Petrecca were previously characterized in our lab [38,39]. GSCs were maintained in neural stem cell complete medium (DMEM/F12, heparin, hEGF, hFGF, penicillin-streptomycin, W21 supplement, and GlutPlus; information provided in Supplementary Table S1). The cells were incubated in a humidified atmosphere of 5% CO_2_ at 37 °C. The cells and GSCs were treated at the indicated concentrations with dimethyl sulfoxide (DMSO; Cat. No. BP231, Fisher Scientific, Fair Lawn, NJ, USA) as a vehicle control, Auranofin (Au; Cat. No. 15316-25, Cayman Chemical Co., Ann Arbor, MI, USA), N-Acetylcysteine (NAC; Cat. No. A9165, Sigma-Aldrich, St. Louis, MO, USA), L-buthionine sulfoximine (L-BSO; Cat. No. B2515, Sigma-Aldrich, Saint Louis, MO, USA), or Piperlongumine (PPL; Cat. No. 528124, Sigma-Aldrich, St. Louis, MO, USA). We generated stable isogenic pairs of p53-knockdown short hairpin RNA (shRNA) OPK49 (OPK49sh) and OPK49 empty vector (OPK49ev) using an shp53 PLKO.1 lentiviral vector (Addgene #19119) [40]. Lipofectamine 3000 was used for the transient transfection of U87MG cells with a plasmid capable of producing a functional p53 protein, following the Invitrogen™ Lipofectamine™ 3000 Reagent Protocol (Invitrogen, Waltham, MA, USA) [41].

### 2.2. MTT Cytotoxicity Assay

The cytotoxic effect of Au was assessed using the Vybrant^®^ MTT Cell Proliferation Assay Kit (Thermo Fisher Scientific Inc., Waltham, MA, USA). Cells were seeded at 2500 cells/well in 96-well plates and allowed to adhere overnight at 37 °C, 5% CO_2_. The cells were then treated with DMSO and various concentrations of Au (0.25–12 µM; for combination purposes, 0.0005–3 µM of Au was combined with 5 µM and 10 µM of L-BSO). After 72 h, the wells were loaded with 10 µL/well of 5 mg/mL MTT [3-(4,5-dimethylthiazol-2-yl)-2,5-diphenyltetrazolium bromide] (Cat. No. M6494, Invitrogen, Carlsbad, CA, USA) in 1× PBS (Cat. No. 311-012, Wisent Inc., Saint-Jean-Baptiste, QC, Canada) solution [42]. The cells were then incubated for 4 h at 37 °C, 5% CO_2_ to allow the tetrazolium dye to reduce to insoluble formazan. Then, 100 µL/well of 10% sodium dodecyl sulfate (SDS)/0.01 M HCl was added to stop the assay [43]. Absorbance was measured at 570 nm using a microplate reader (Bio-Tek Cytation 3 Multi-Mode Reader, Serial No. 131106B, Agilent, Santa Clara, CA, USA) following 18 h of incubation. Cell viability relative to the control was calculated as a percentage after subtracting the absorbance from blank controls.

### 2.3. AlamarBlue Viability Assay

GSC viability was assessed using the alamarBlue kit (Thermo Fischer Scientific, Waltham, MA, USA). Briefly, GSC neurosphere cultures were dissociated with Accumax (Millipore, Burlington, MA, USA), seeded in triplicate in 96-well plates, and allowed to form spheres in neural stem cell complete medium over 48 h. Next, varying concentrations of Au (0.25–12 µM) or the DMSO control were added in triplicate and incubated for an additional 5 days. For combination purposes, 0.0005–3 µM of Au was combined with 5 µM or 10 µM of L-BSO. AlamarBlue was added to each well, incubated for 6 h at 37 °C, and the absorbance was recorded using a Gemini XPS fluorescence microplate reader (540EX/600EM nm, Santa Clara, CA, USA) [44]. GSC viability relative to the control was calculated as a percentage after subtracting the absorbance from the blank controls.

### 2.4. Clonogenic Assay

U87MG and T98G cell lines were trypsinized during the exponential growth phase and 3 × 10^2^ single-cell suspensions were seeded in triplicate in complete medium in 6-well plates and incubated to adhere overnight at 37 °C, 5% CO_2_. The next day, the medium was replaced with the DMSO control or the drug-containing medium and the cells were further incubated at 37 °C for 9–11 days. The cells were then fixed with 10% formalin, stained with 0.05% crystal violet, and colonies containing more than 50 cells were counted. The surviving fraction was normalized to the plating efficiency of the DMSO control for each cell line using the formula: (number of colonies/number of cells plated)/(plating efficiency of DMSO-treated control cells) × 100 [45].

### 2.5. Neurosphere Formation Assay

The ability of GSCs to form spheres was examined using neurosphere self-renewal assays [46]. Briefly, cells were seeded in neural stem cell complete medium at a concentration of 5000 cells/well in ultra-low attachment 6-well plates. Au at 0.5 µM was added to each well and DMSO was used as a control. For the combination experiments, 0.01 µM of Au was used in combination with 1 mM of NAC or 1 µM of L-BSO. After 14–20 days the total number of neurospheres in each well was counted and normalized to the DMSO control.

### 2.6. Western Blot Analysis

Cells were seeded overnight in standard medium or in neural stem cell complete medium for GSCs before treatment (drug or control) for the time and concentration shown. The cells were rinsed with cold PBS, scraped, collected, and centrifuged at 271× *g* for 5 min at 4 °C. The GSCs were centrifuged and washed with cold PBS. The supernatant of cells or GSCs was removed, and the cell pellet was resuspended in lysis buffer by gentle vortexing, then lysed with RIPA buffer (Boston BioProducts, Milford, MA, USA) supplemented with sodium orthovanadate protease (Sigma-Aldrich, Saint Louis, MO, USA) and phosphatase inhibitor cocktails (Roche Diagnostics, Basel, Switzerland). The lysates were then placed on ice in a shaker at 4 °C for 30 min and centrifuged at 18,440× *g* for 10 min at 4 °C. The lysate supernatant was removed to determine the protein concentrations in each sample using a Pierce™ BCA Protein Assay Kit (Thermo Fisher Scientific, Waltham, MA, USA). Electrophoretic separation of samples and immunoblotting to detect target proteins were performed using the following primary antibodies: p-AKT/Ser473 (193H12), AKT, P-ERk1/2, ERK1/2, TrxR1 (B-2), PARP-1, p53, and β-Actin (Antibodies concentration and information are provided in Supplementary Table S1), as previously described by our group [39,40]. Following incubation with appropriate secondary antibodies, the membranes were analyzed using chemiluminescence Clarity Western ECL imaging (BioRad Laboratories Inc., Hercules, CA, USA) and the ChemiDoc imaging system (BioRad Laboratories Inc., Hercules, CA, USA). Densitometry analysis was performed using Image Lab 6.1 Software. Each treatment condition was normalized to the actin loading control and compared against the DMSO (control) for each GSC.

### 2.7. Measurement of ROS Production

To assess ROS levels after treating cells with Au, CM-H2DCFA (5-(and 6-)-chloromethyl-2′,7′-dichlorodihydrofluoresceine diacetate; Cat. No. C6827, Invitrogen, Carlsbad, CA, USA) was used. Cells were seeded in 96-well plates and allowed to either adhere overnight or form spheres for 72 h. Different drug concentrations were added in triplicate. The cells were then incubated for 24 h at 37 °C. Next, 10 µM of CM-H2DCFA was diluted in clear media and added to cells for 30 min at 37 °C. Next, the cells were loaded with 5 µM Hoechst Solution (20 mM; Cat. No. 62249, Thermo Fischer Scientific, Waltham, MA, USA) to stain the nuclei and determine cell count, followed by the measurement of absorbances using a fluorescent microplate reader at an excitation of 490 nm and emission of 525 nm for the ROS probe and an excitation of 361 nm and emission of 497 nm for the Hoechst probe (Tecan, Infinite M200, Mannedorf, Switzerland). Relative ROS production was calculated using ROS fluorescence intensity normalized to Hoechst fluorescence intensity followed by use of the following equation: (F_drug treated_ − F_blank_)/(F_control_ − F_blank_).

### 2.8. Gene Expression and Correlation Analysis in Publically Available Human Datasets

Microarray data from the following publicly available databases were utilized: The Cancer Genome Atlas (TCGA; https://www.cancer.gov/tcga, accessed on 7 September 2023) Firehose Legacy and Cell 2013 databases consisting of 528 and 153 patient samples, respectively, which were sourced from cbioportal.com (accessed on 7 September 2023). The data consisted of mRNA expression levels, which were measured using U133 microarray and RNAseq vs. RSEM. The normalization of mRNA expression data was achieved through log2 transformation. To dissect the influence of *TP53* alterations and their correlations with *Txnrd1,* the patient cohort was stratified into two groups: those with *TP53* alterations (72 patients) and those without (524 patients). Spearman’s and Pearson’s rank correlation coefficients were employed to measure the strength and direction of association between *Txnrd1* and GSH system genes across the various subgroups. The datasets were accessed and analyzed for gene expression using the GlioVis web application (http://gliovis.bioinfo.cnio.es, accessed on 31 August 2024) [47]. Correlation AnalyzeR was applied to assess possible correlation co-expression and pathways enrichment between two genes of interest in normal and tumor samples (https://gccri.bishop-lab.uthscsa.edu/, accessed on 31 August 2024).

### 2.9. Statistical Analysis

GraphPad Prism (GraphPad Software Inc., La Jolla, CA, USA) was used for statistical analysis. Data are reported as mean ± SEM and are representative of at least three independent experiments unless otherwise stated. One-way ANOVA for the analysis of one independent variable or two-way ANOVA for two independent variables followed by post hoc multiple comparisons testing were performed for comparisons involving three or more groups, and *p* values < 0.05 were considered statistically significant. SynergyFinder+ (https://synergyfinder.org/, accessed on 7 August 2023) was used to find the synergy score of the Au combinations with L-BSO or PPL. Scores > 10 are considered synergistic, 0 to 10 are considered additive, and lower than 10 are considered antagonistic.

## 3. Results

### 3.1. Au Decreased Viability of GBM Cell Lines and GSCs, with Enhanced Sensitivity in p53-Knockdown GSCs

**Figure 1:**
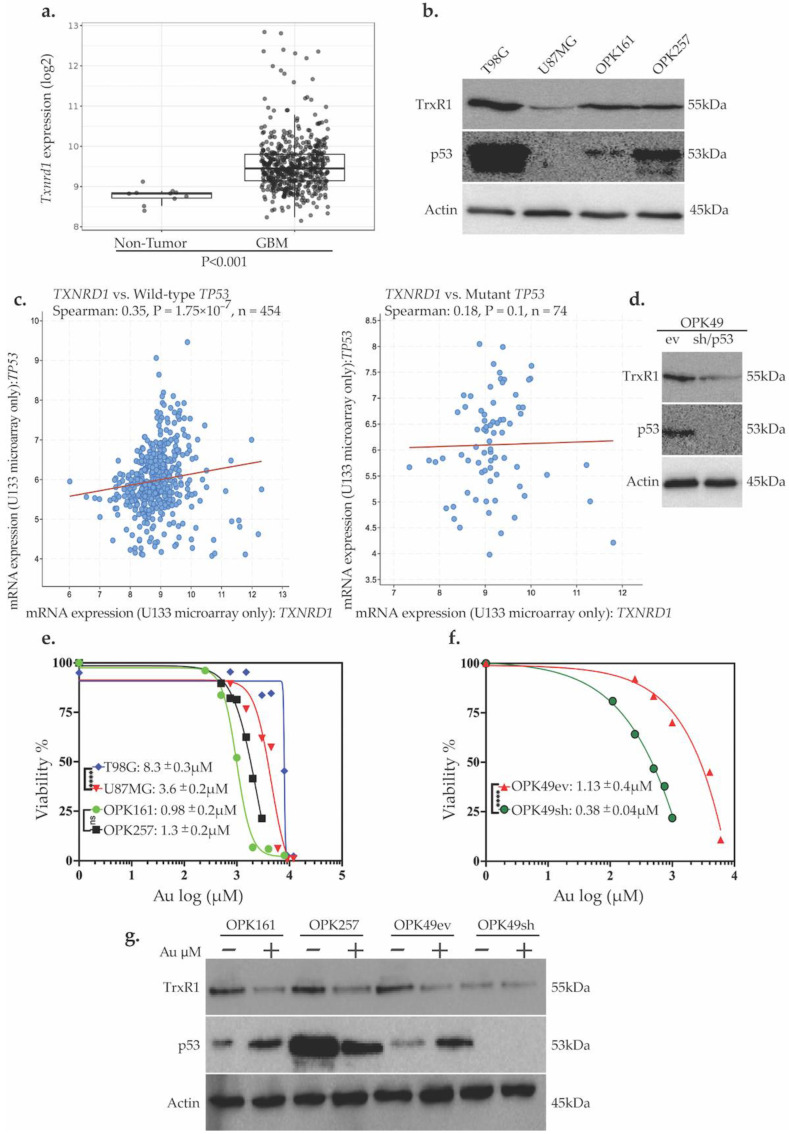
**Au decreases viability of GBM cell lines and GSCs, with enhanced sensitivity in a p53-knockdown GSC line and decreased TrxR1 protein expression in GSCs**. (**a**) TCGA analysis of *Txnrd1* encoding for TrxR1 (*y*-axis: *Txnrd1* mRNA expression, *x*-axis: sample types, pairwise *t*-tests, box plot shows the SEM). (**b**) Western blot analysis of TrxR1 and p53 protein expression at basal level. (**c**) Spearman correlation between *Txnrd1* and wild-type *TP53* (**left**) and between *Txnrd1* and mutant *TP53* (**right**) (The Cancer Genome Atlas [TCGA], Firehose Legacy). (**d**) Western blot analysis of TrxR1 expression in the GSC OPK49 empty vector (OP49ev), control, which harbors wild-type p53, and its counterpart p53-knockdown OPK49shRNA (OPK49sh). (**e**,**f**) Au cytotoxicity (*y*-axis: viability%, *x*-axis: log10 of Au concentrations, IC50s: µM, **** *p* < 0.0001) using alamarBlue assay of GSCs treated for 5 days and MTT assay of GBM cell lines treated for 72 h. (**g**) Western blot analysis of TrxR1 and p53 expression post-Au treatment (1 µM, 24 h). Actin was used as a loading control.

To assess the potential relevance of TrxR1 as a target in GBM, we performed TCGA analysis for the expression of *Txnrd1*, the gene that encodes TrxR1, in newly diagnosed GBM patients (528 GBM samples compared to 10 normal samples). Given the role of p53 as a redox-sensitive transcription factor in ROS regulation [23], we analyzed *Txnrd1* mRNA expression in GBM tumor tissues based on their wild-type or mutant (mut) *TP53* status. Our analysis revealed that *Txnrd1* mRNA (the gene encoding for TrxR1) is significantly overexpressed in GBM tumor tissues compared to normal tissue (*p* < 0.001) (Figure 1a). We further assessed TrxR1 and p53 expression by western blot analysis in the GBM cell lines T98G (mutp53) and U87MG (wtp53) and the patient-derived GSCs OPK161 (wtp53) and OPK257 (mutp53) (Figure 1b).

As a transcription factor, p53 regulates genes involved in the redox system [22,24]. Therefore, we also investigated the potential relationship between p53 and TrxR1 using the TCGA GBM dataset. A significant positive correlation was found between *Txnrd1* and wt*TP53* mRNA using the TCGA Firehose Legacy dataset for 454 GBM patients (n = 454, R = 0.35, *p* = 1.75 × 10^−7^), but no such correlation was found between *Txnrd1* and mut*TP53* (n = 74, *p* = 0.1) (Figure 1c). RNA sequencing analysis (*Cell* 2013 database) showed a significantly higher correlation compared to mRNA microarray analysis (n = 152, R = 0.51, *p* < 0.001). The GlioVis web application AnalyzeR showed a higher correlation for normal brain samples (n = 6397, R = 0.86, *p* < 0.01) and for brain tumor samples which included ~50% of GBM samples (n = 5501, R = 0.69, *p* < 0.01).

To assess the relationship between p53 status and TrxR1, we used a short hairpin (sh)RNA silencing approach in the wtp53 GSC control—the OPK49 empty vector (OPK49ev)—to produce its counterpart harboring p53-knockdown (OPK49sh) [38,39]. Interestingly, p53-knockdown in OPK49sh decreased TrxR1 expression by at least 90% compared to OPK49ev (Figure 1d). On the other hand, we increased p53 expression by generating a transient transfection of *TP53* in U87MG with a low p53 expression and found increased TrxR1 expression (Figure S1). These results revealed a positive relationship between wtp53 and TrxR1 in a GSC, a GBM established cell line, and the GBM/TCGA dataset.

We assessed the viability of patient-derived GSCs and GBM cell lines using Au, a pan-TrxR inhibitor. Treatment with various Au concentrations ranging between 0.25–12 µM revealed a higher sensitivity of GSCs to Au in comparison to GBM cell lines, with non-significant differences in IC50 values within a 1 µM range (*p* > 0.05) among GSCs (OPK161: 0.98 ± 0.2 µM, OPK257: 1.14 ± 0.2 µM, OPK49ev: 1.13 ± 0.4 µM) but higher IC50 values for GBM cell lines (T98G: 8.5 ± 0.3 µM and U87MG: 3.6 ± 0.2 µM) (Figure 1e). The positive correlation between p53 and TrxR1 prompted our interest in testing the role of p53 in response to Au. Interestingly, the p53-knockdown GSC OPK49sh showed higher sensitivity to Au (IC50: 0.38 ± 0.04 µM) than its counterpart OPK49ev (*p* = 0.0001) (Figure 1f). As shown by western blotting analysis, Au decreased TrxR1 expression levels in all GSCs. It also increased p53 expression in wt-p53 OPK161 and OPK49ev GSCs. Interestingly, Au decreased the expression of mutp53 in OPK257 (Figure 1g). These results highlight Au’s cytotoxicity in GSCs compared to GBM cell lines and unravel the role of wtp53 as a negative redox-sensitive mechanism in response to Au in GSCs isogenic for p53.

### 3.2. Au Induces ROS-Dependent Long-Term Cytotoxicity in GSCs and GBM Cell Lines with p53-Knockdown GSC Line Displaying the Highest ROS Increase

**Figure 2:**
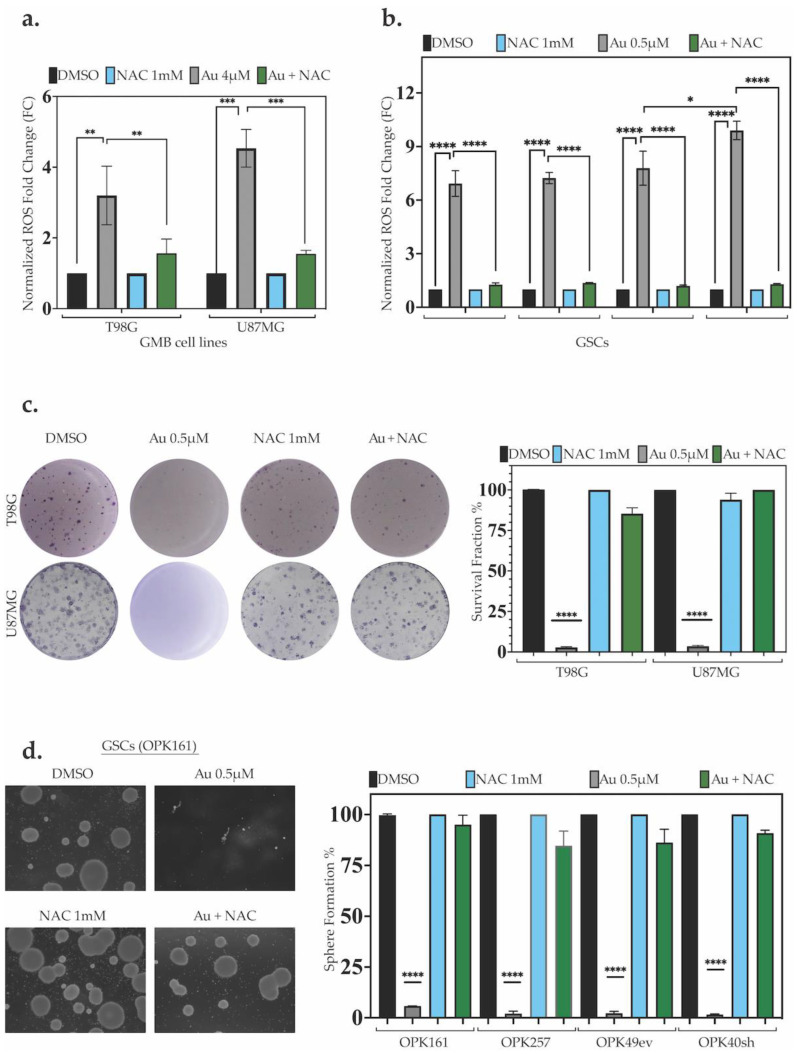
**Au induces ROS-dependent long-term cytotoxicity in GSCs and GBM cell lines**. (**a,b**) Intracellular ROS fold change of relative fluorescence units (RFU) for (**a**) T98G and U87MG cells treated with DMSO, 4 µM Au, 1 mM NAC alone or in combination and (**b**) OPK161, OPK257, OPK49ev, and OPK49sh GSCs treated with 0.5 µM Au, 1 mM NAC alone, or in combination for 24 h. (**c**) Representative clonogenic assay images of T98G and U87MG cells and graph for surviving fractions of cells treated with 0.5 µM Au, 1 mM NAC alone, or in combination for 10–14 days. (**d**) Neurosphere formation of GSCs treated with DMSO, 0.5 µM Au, 1 mM NAC alone, or in combination for 20 days. Representative images are shown with scale bar 200 µM. Spheres over 50 µM in size were counted under a microscope (20× magnification). Bar charts show the SEM (* *p* < 0.05 ** *p* < 0.01 *** *p* < 0.001 **** *p* < 0.0001).

To assess the effect of Au on ROS generation, we exposed GSCs and GBM cell lines to Au at concentrations of 0.5 µM for GSCs and 4 µM for cell lines over 24 h. The fluorescent ROS-sensing probe CM-H2DCFDA was used to measure the levels of ROS. Au induced a substantial and significant rise in ROS (3.2- to 9.9-fold increase), with the highest elevation observed in GSCs, specifically in OPK49sh, and the lowest in T98G (Figure 2a,b). To assess the role of ROS in Au-induced cytotoxicity, we used N-acetylcysteine (NAC), a precursor of intracellular cysteine and glutathione that scavenges free radicals either directly via the redox potential of thiols or indirectly by increasing the glutathione levels in cells [48]. NAC significantly prevented Au-induced ROS increase in both cell types and to the same extent (Figure 2a,b).

Next, we evaluated whether ROS elevation induced by Au could induce long-term cellular toxicity after treating the cells with a low concentration of 0.5 µM Au (a single exposure on day 1) and incubating for 14–20 days. Remarkably, this low dose of Au almost completely suppressed the clonogenic survival fraction of the GBM cell lines T98G and U87MG (Figure 2c). It also drastically depleted the sphere-forming capabilities of GSCs (Figure 2d). Interestingly, the combination of Au 0.5 µM and NAC 1 mM completely prevented Au cytotoxicity, and the cells maintained their ability to grow, similar to the DMSO control condition (Figure 2c,d). These results demonstrate that Au-induced ROS significantly decreases the survival of GSCs and GBM cell lines and mediates ROS-dependent short-term and long-term cytotoxic effects.

### 3.3. Correlation of GSH-Metabolizing Enzymes with Txnrd1 in GBM Datasets and Synergistic Cytotoxicity of Auranofin and L-BSO, a GSH Inhibitor

**Figure 3:**
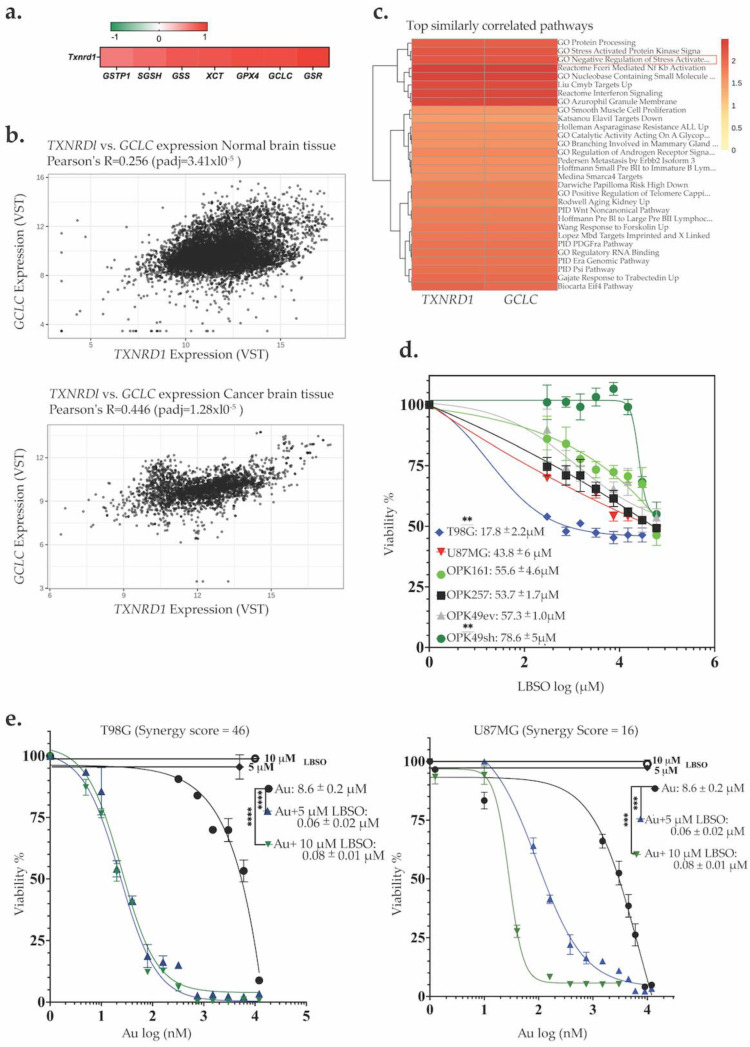

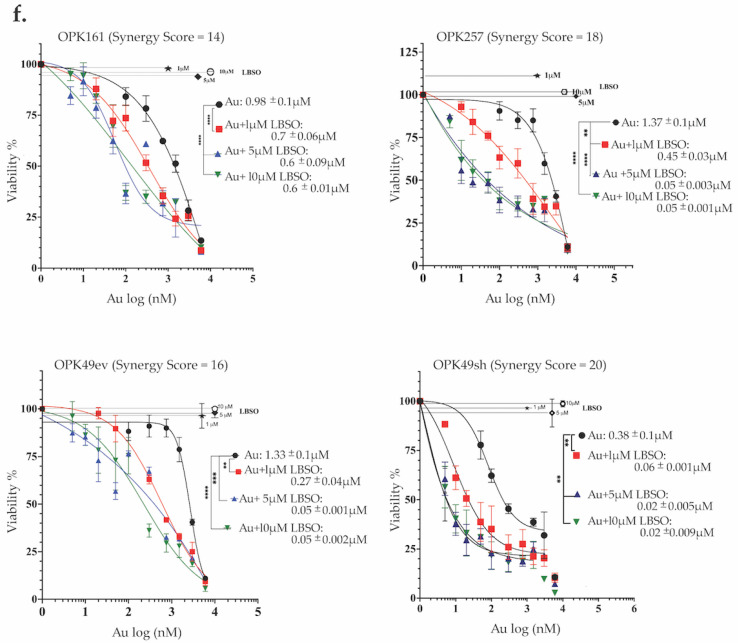
**ROS-inducer compound L-BSO, a GSH inhibitor, enhances Au cytotoxicity**. (**a**) Summarized correlation among GSH system members (GSR, GSS, SGSH, XCT, GPX4, GCLC and GSTP1) and *Txnrd1* in 152 samples with available data from the TCGA GBM cohort of 577 patients. Correlation graphs are provided in Figure S3. (**b**) Co-expression correlation of *Txnrd1* and *GCLC* RNA-sequencing read counts in normal and brain cancer tissue. (**c**) Heatmap showing the top similarly correlated pathways with both GCLC and *Txnrd1*. (**d**) Dose-response curve of GBM cell lines and GSCs treated with increasing doses of L-BSO (1–100 µM) for 72 h or 5 days. (**e**,**f**) Dose-response curve of GBM cell lines and GSCs treated with varying concentrations of Au and co-treatment with 5 µM or 10 µM L-BSO (GBM cell lines T98G and U87MG) or 1, 5, or 10 µM L-BSO (GSCs). Cell viability was assessed using MTT and alamarBlue assays for GBM cell lines and GSCs, respectively. Scores > 10 are considered synergistic, 0 to 10 are considered additive, and lower than 10 are considered antagonistic. GraphPad Prism was used to generate the graphs and determine IC50 values (** *p* < 0.01 *** *p* < 0.001 **** *p* < 0.0001).

The GSH system often compensates for the absence of other antioxidant mechanisms by working in tandem with the Trx system. We analyzed the correlation between TrxR1 and the GSH system in GBM patients using RNAseq vs. RSEM (RNA-seq by Expectation-Maximization estimating gene and isoform expression levels from RNA-seq data) data available for 153 samples in TCGA, Cell 2013. Our analysis unveiled a positive correlation between TrxR1 and key proteins involved in biosynthesis, recycling, and membrane transportation in the GSH system at the mRNA level. *Txnrd1* exhibited significantly strong correlations (R ≥ 0.7, *p* < 0.0001) with glutathione-disulfide reductase (*GSR*), gamma-glutamate-cysteine ligase catalytic (*GCLC*), *GSS*, *SGSH*, *XCT*, *GPX4*, and glutathione S-transferase pi-1 (*GSTP1*) (R = 0.5–0.69, *p* < 0.0001) (Figure 3a and corresponding graphs in Figure S3).

We found that *GCLC*, a rate-limiting enzyme of glutathione synthesis, was among the GSH-metabolizing enzymes with the highest significant positive correlation with *Txnrd1* (R = 0.7, *p* = 5.46 × 10^−21^). We subsequently utilized the Correlation AnalyzeR web application to generate a scatter plot analysis of *GCLC* and *Txnrd1* co-expression in normal and brain cancer tissue. We identified a significant increase in *GCLC* and *Txnrd1* co-expression in brain cancer tissue (Pearson R = 0.44, *padj* = 1.28 × 10^−5^) compared to normal brain tissue (Pearson R = 0.25, *padj* = 3.41 × 10^−5^) (Figure 3b). Additionally, enrichment analysis and heat maps of top similarly correlated pathways confirmed both genes as negative regulators of stress activation (Figure 3c). The positive correlation between *Txnrd1* and *GCLC* in two independent databases guided our hypothesis that co-targeting TrxR1 and GCLC might be required to overwhelm GBM cells with lethal levels of ROS.

L-BSO inhibits GCLC, leading to GSH depletion [49,50]. Therefore, we sought to assess the potential synergistic effect of Au combined with L-BSO. Prior to combination, we first assessed L-BSO toxicity alone. Both GSCs and GBM cell lines exhibited lower sensitivity (higher IC50s) to L-BSO alone compared to Au alone. The L-BSO IC50 values demonstrated considerable variation, with the highest sensitivity observed in T98G at 17.8 µM and the lowest sensitivity in OPK49sh at 78.6 µM (Figure 3d). For U87MG, OPK161, OPK257, and OPK49ev, the IC50 values were 43 µM, 55 µM, 53 µM, and 57 µM, respectively.

Next, we combined Au and L-BSO using a range of Au concentrations from 0.005–3 µM, combined with either 5 or 10 µM of L-BSO for GBM cell lines or 1 µM or 5 µM for GSCs. Our results demonstrate that L-BSO significantly increased the cytotoxicity of Au in both GBM cell lines and GSCs, leading to a notable reduction in Au IC50 values to nanomolar ranges (Figure 3e,f). In GBM cell lines, both concentrations of L-BSO (5 and 10 µM) decreased Au IC50s to, respectively, 0.1 and 0.06 µM for U87MG and 0.08 and 0.06 µM for T98G. In OPK161, OPK257, OPK49ev, and OPK49sh GSCs, 1 µM of L-BSO combined with Au decreased the IC50 of Au to 0.17 µM, 0.45 µM, 0.27 µM, and 0.06 µM, respectively. Increasing the concentration of L-BSO to 5 and 10 µM enhanced efficacy up to a plateau (decreased Au IC50s similarly to the range of 0.07–0.02 µM), beyond which no further improvements in the effects was observed (decrease in Au IC50 values). Using the SynergyFinder Plus web tool (https://synergyfinder.org) [51], we explored the synergistic effects of L-BSO and Au as a combined treatment. This tool applies four different algorithms: ZIP (zero interaction potency), HSA (highest single agent), Bliss (Bliss independence), and Loewe (Loewe additivity) to determine the synergistic effect as the excess of the observed effect over the expected effect calculated by reference models (synergy scoring models) [52,53]. To increase the robustness of the analysis, we confirmed the synergy between the two drugs only if the four algorithms showed global positive results with a synergy score (SC) > 10 considered synergistic, while SC (0–10) is considered additive. (Figure S4) [54]. Intriguingly, the highest synergy score (SC) was observed in T98G (SC = 46), while U87MG exhibited a relatively lower synergistic response (SC = 16). Notable synergistic scores (>10) were also recorded for OPK49ev (SC = 16), OPK49sh (SC = 20), OPK257 (SC = 18), while OPK161 displayed the lowest synergistic response (SC = 14) (Figure S4).

### 3.4. Combining Au with L-BSO Synergistically Increased ROS and Long-Term Cytotoxicity Compared to Each Drug Alone in GBM Cell Lines and GSCs

**Figure 4:**
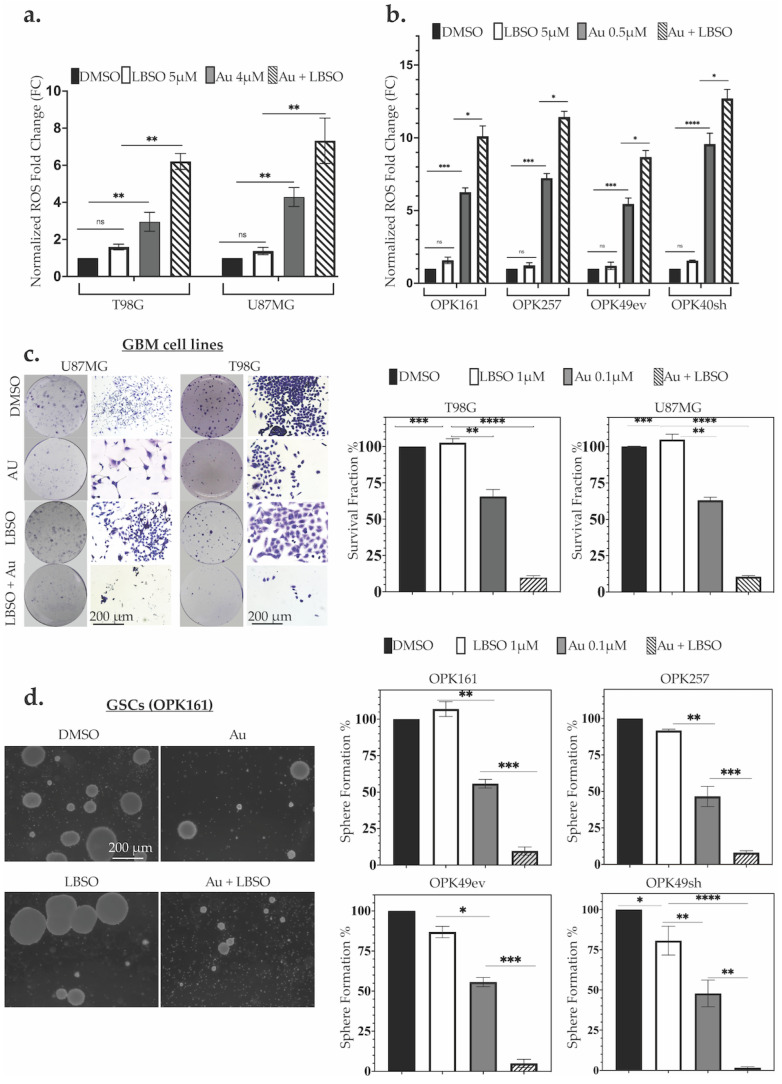
**Combining Au with L-BSO resulted in significantly higher ROS levels and long-term cytotoxicity compared to each drug alone.** (**a**) ROS elevation in T98G and U87MG GBM cell lines after 24 h treatment with 4 µM Au and/or 5 µM L-BSO. (**b**) ROS elevation in OPK161, OPK257, OPK49ev, and OPK49sh GSCs following 24 h treatment with 1 µM Au and/or 5 µM L-BSO. ROS absorbance was normalized to DMSO controls. (**c**) Clonogenic assays of T98G and U87MG following treatment with 0.1 µM Au and/or 1 µM L-BSO. Survival fraction was calculated after fixing and staining with crystal violet. (**d**) Neurosphere formation in GSCs evaluated upon treating cells with 0.1 µM Au and/or 1 µM L-BSO. After 20 days, spheres were counted under a microscope (20× magnification). Bar charts show the SEM (* *p* < 0.05 ** *p* < 0.01 *** *p* < 0.001 **** *p* < 0.0001).

We did not observe any significant increase in ROS levels when GBM cell lines and GSCs were treated with L-BSO alone compared to their respective controls (Figure 4a,b). Combining 5 µM L-BSO with 1 µM Au for 24 h resulted in a significant increase in ROS levels compared to Au alone, underlining the synergistic effect of Au and L-BSO in both GBM cell lines and GSCs (Figure 4a,b).

To assess the long-term effects of Au, L-BSO, and combinations of the two, we performed neurosphere formation and clonogenic assays for GSCs and the GBM cell lines, respectively. While Au alone at 0.1 µM decreased neurosphere and colony formation in both GSCs and GBM cell lines to 50–60%, 1 µM of L-BSO alone did not induce significant cytotoxicity in the cell lines (Figure 4c). However, Au at 0.1 µM combined with L-BSO at 1 µM induced drastic long-term cytotoxicity in GBM cell lines and GSCs. The survival fractions of GBM cell lines were reduced to 2.7% and 3.4% for T98G and U87MG, respectively. Neurosphere formation was inhibited to 5.8%, 2.0%, 2.3%, and 1.6% for OPK161, OPK257, OPK49ev, and OPK49sh, respectively (Figure 4c,d). Combining Au at only 0.1 µM with L-BSO induced synergistic long-term cytotoxicity compared to each drug alone in GBM cell lines and GSCs.

### 3.5. Combination of Au with L-BSO Decreased Cellular Survival Pathways and Induced Apoptosis in GSCs

**Figure 5:**
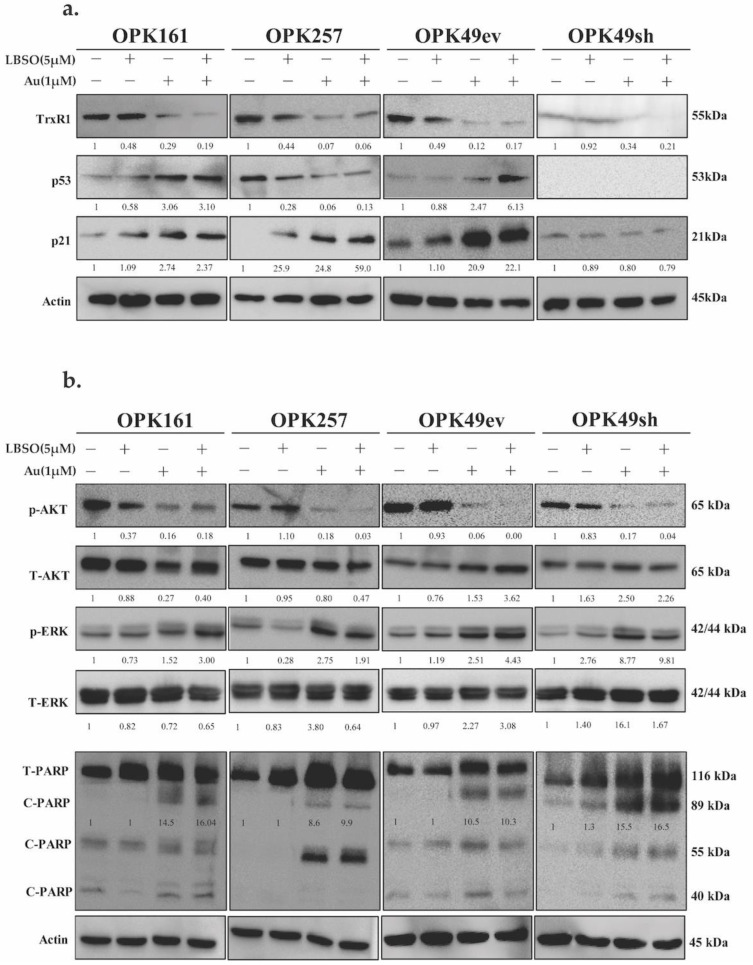
**Au alone and in combination with L-BSO decreased TrxR1 and P-AKT while inducing wtp53 activation and apoptosis in GSCs.** Western blot analysis showing expression of (**a**) TrxR1, p53, p21 and (**b**) phosphorylated p-AKT (Ser-473), total (T-AKT), phosphorylated ERK1/2 (p-ERK), total ERK1/2 (T-ERK), total (T-PARP-1) and cleaved (C-PARP) apoptotic bands (89 kDa) and necrotic bands (55 kDa). wtp53 OPK161, mutp53 OPK257, wtp53 OPK49ev, and wtp53-knockdown OPK49sh GSCs were treated with DMSO control (−) or 1 µM (+) Au and/or 5 µM L-BSO (+) for 24 h. Actin was used as a loading control. Band intensities were quantified and normalized to actin. Values are shown relative to DMSO control. Bar graphs of densitometry analysis are provided in Supplementary Figure S5.

Previous studies have highlighted the role of ROS in activating p53 and apoptotic pathways [21]. In this study, we investigated the impact of L-BSO, Au, and combinations of the two on TrxR1 and p53 expression in GSCs. We found that while L-BSO did not change TrxR1 expression, the addition of Au decreased TrxR1 expression in all GSCs (Figure 5a). Moreover, we examined the effects of Au and L-BSO alone or in combination on p53 activation in GSCs harboring wtp53 (OPK161 and OPK49ev). Interestingly, both Au alone and Au in combination with L-BSO induced p53 activation and the upregulation of its downstream target, p21, in both of these cell lines (Figure 5a). In OPK257, mutp53 expression exhibited a modest decrease following L-BSO treatment, but a significant reduction was observed after treatment with Au alone and Au in combination with L-BSO, accompanied by an induction of p21 expression (Figure 5a).

We extended our investigation to assess the effect of L-BSO, Au, and combinations of the two on GSCs by examining the activation of key proteins involved in cell signaling pathways and apoptosis [38,55]. We analyzed the AKT and ERK signaling pathways due to their pivotal involvement in cell survival and proliferation. Across all GSCs, the treatment using Au alone or combining L-BSO and Au resulted in a significant reduction in p-AKT (Ser-473) while the levels of total AKT (T-AKT) were nearly constant across all conditions (Figure 5b). ERK phosphorylation was observed in response to Au treatment and Au in combination with L-BSO, while total ERK levels remained unchanged. Next, we assessed the activation of apoptotic/necrotic PARP-1 [56,57] and observed PARP-1 cleavage bands at 89 kDa and necrotic cleavage bands (55 kDa) in Au alone and in combination with L-BSO. OPK257 showed a higher cleavage of necrotic PARP bands (55 kDa) compared to apoptotic cleavage fragments. It is worth mentioning that a lower basal level of PARP was observed in OPK49sh. While L-BSO alone did not substantially affect AKT, ERK, phosphorylation, or PARP when compared to Au alone, its combination with Au did not increase the molecular effects of Au for the time and concentration analyzed in GSCs (Figure 5b and Figure S5).

### 3.6. Auranofin Increased GSTP-1 While Piperlongumine (PPL) Induced Significant Cytotoxicity and a Strong Synergistic Effect Within a Nanomolar Range in GSCs

**Figure 6:**
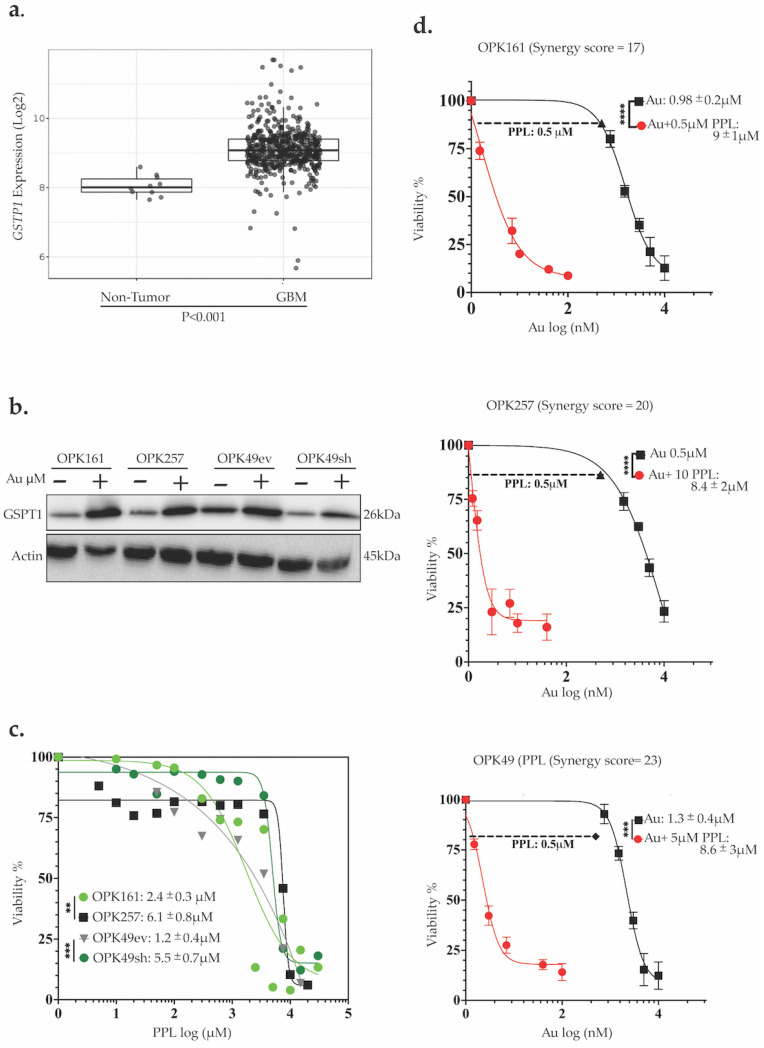
**Auranofin (Au) increased GSTP-1 and synergistically increased Piperlongumine (PPL) cytotoxicity to a nanomolar range in GSCs**. (**a**) TCGA analysis of GSTP1 expression in 528 GBM patients compared to 10 normal samples (*y*-axis: *Txnrd1* expression, *x*-axis: sample types, pairwise *t*-tests, bar charts show the SEM). (**b**) Western blotting analysis of GSTP1 expression in GSCs following Au treatment at 1 µM for 24. (**c**) Evaluation of PPL cytotoxicity (*y*-axis viability%, *x*-axis log10 of Au concentrations, IC50s: µM, **** *p* < 0.0005) using alamarBlue assay in GSCs treated for 5 days (**d**) Dose-response curves of GSCs treated with varying Au concentrations and co-treatment with 0.5 µM PPL. IC50 values were determined, and graphs were generated using GraphPad Prism (** *p* < 0.01 *** *p* < 0.001 **** *p* < 0.0001).

Given the significant synergistic effect observed with Au and L-BSO and the toxicity of L-BSO in the clinic [58], we also tested PPL, a natural product reported to exhibit anticancer activity and inhibit the GSH system by targeting GSTP1 [59]. To explore the relevance of GSTP-1 expression in GBM, we analyzed *GSTP-1* mRNA expression in normal brain tissue and GBM patients from the TCGA GBM patient dataset. GBM TCGA data analysis showed that GSTP1 is significantly overexpressed in GBM patients (Figure 6a). Interestingly, GBM/TCGA analysis also revealed a positive correlation between GSTP1 and TrxR1, the target of Au (Figure 3a). Next, we investigated whether Au treatment might affect GSTP1 expression. Au at 1 µM for 24 h increased the expression of GSTP1 in all GSCs, potentially as a compensatory mechanism in response to the inhibition of TrxR1 (Figure 6b). We then treated GSCs with various concentrations of PPL, ranging from 1–25 µM, and determined the IC50 values using an alamarBlue assay. Our results showed that PPL alone significantly decreased the viability of GSCs, with IC50 values of 2.4 ± 0.3 µM for OPK161 and 6.1 ± 0.8 µM for OPK257 (Figure 6c). Notably, while wt-p53 knockdown in OPK49sh heightened sensitivity to Au (Figure 1f), wt-p53 knockdown in OPK49sh decreased sensitivity to PPL, i.e., increased the IC50 (5.5 ± 0.7 µM) compared to the IC50 (1.2 ± 0.4 µM) of the control OPK49ev (Figure 6c). We also tested the potential synergistic effect of Au with PPL in three of the GSCs. A remarkable synergistic effect was observed, with a significant decrease in Au IC50 values to nanomolar ranges (0.8 nM in both OPK161 and OPK257, and 8.6 nM in OPK49) (Figure 6d). Synergistic scores (>10) were recorded for the combined treatment of Au and PPL in OPK161 (SC = 17), OPK49 (SC = 23), and OPK257 (SC = 20) (Figure S5).

## 4. Discussion

Treatment of GBM poses a daunting challenge due to its aggressive nature and therapeutic resistance, often attributed to the elevated expression and/or activity of the Trx and GSH antioxidant systems [8,60]. In this study, we unraveled the vulnerabilities of GSCs related to a pro-oxidant Trx and/or GSH inhibition strategy in GBM cell lines and GSCs, a subpopulation notorious for their therapeutic evasion [61]. We made new findings related to the role of p53 and the sensitivity of GSCs to Au alone and in combination with a GSH-targeting strategy in GBM: *(i)* We identified a protective role of wtp53 and the potential role of wtp53-TrxR1 axis in response to Au in GSCs. *(ii)* We determined the onset of ROS-scavenging adaptive mechanisms, such as increased GSTP1 following Au treatment, and the high significant positive correlation between TrxR1 and 7 GSH system components in the TCGA datasets of patients newly diagnosed with GBM, in addition to *(iii)* the ability of pro-oxidant Trx/GSH inhibitors to bypass redox compensatory mechanisms in GSCs and exhibit strong cytotoxic synergy regardless of wtp53 expression levels and mutant-p53 in GSCs. ROS is known to play a dual role in GBM, driving GSC resistance mechanisms and tumor progression at moderate levels, while a pro-oxidant strategy inducing excessive ROS levels can be detrimental to their survival [17]. Our recent findings show the superior efficacy of Au combined with L-BSO compared to Au alone in EGFR-positive U87MG GBM cell lines [62]. The current study revealed the synergistic response irrespective of *TP53* status in GSCs, the role of GSTP1 as a potential antioxidant compensatory mechanism, and the subsequent efficacy of PLL in combination with Au within a nanomolar range in GSCs. The synergistic increase of ROS levels to cytotoxic lethal levels in response to co-targeting the Trx and GSH systems in GBM cell lines and GSCs might be required to overcome their intrinsic resistance to pro-oxidant strategies.

Our study reveals for the first time the role of wtp53 in the positive regulation of TrxR1 protein expression in the wtp53-knockdown GSC model (Figure 1d,g). Conversely, transient transfection of U87MG cells with wt-p53 elevated TrxR1 protein levels (Figure S2), suggesting a potential positive regulation of TrxR1 by wtp53 in U87MG cells. We showed that wtp53 negatively affects the response to Au, as demonstrated by a significantly higher sensitivity to Au and increased ROS production in knockdown-p53 GSCs compared to its counterpart. These findings substantiate the importance of targeting TrxR1 by showcasing Au cytotoxicity against GSCs and revealing the protective role of wt-p53 against Au, potentially through transcriptional regulation of *Txnrd1* encoding for TrxR1 by p53. The gene *GSTP1* has been shown to be a direct downstream transcriptional target of wtp53. Small interfering RNA-mediated reduction of p53 expression specifically decreased GSTP1 expression. While wtp53 transcriptionally regulates *GSTP1* as a potential mechanism to protect the genome [28], the relationship between TrxR1 as a downstream transcriptional target of wtp53 remains to be investigated.

The Trx system, particularly TrxR1, has emerged as a critical factor in GBM prognosis and drug resistance [9,63], reflecting its potential as a therapeutic target. Among other TrxR inhibitors, Au stands out as a repurposed, well-tolerated TrxR1 inhibitor with blood-brain barrier penetration [64], neuroprotective effects [35], and a potential for fast-track clinical translation. Importantly, Au has been evaluated in clinical trials for several cancers, including GBM in the CUSP9 (coordinated undermining of survival paths by 9 repurposed drugs) clinical trial for recurrent GBM [65]. The mechanism of action of Au is primarily mediated through the generation of ROS, an outcome corroborated by our findings that the ROS scavenger, NAC, reverted Au cytotoxicity. Au binds directly to and inhibits its main targets: cytosolic TrxR1 and mitochondrial TrxR2 [59,66].

In this study, we used two GBM cell lines, U87MG and T98G, to represent wild type and mutant TP53, respectively. In a previous study, we sequenced *TP53* to confirm that the T98G cell line exhibits the *TP53* mutation in the DNA-binding domain of p53 protein (M237I substitution, reported as a gain-of-function mutation), and we confirmed wtp53 status for U87MG. We also used one GSC with mutp53 (OPK257), which showed a high expression of p53 protein in western blotting and strong expression of p53 in an immunohistochemistry analysis in the corresponding patient pathology report [38]. In the current study, we validated our findings in 2 GSCs with wtp53 (OPK161 and OPK49) and used TCGA to analyze the correlation between *Txnrd1* and wtp53 or mutant *TP53* in GBM patients’ datasets. We acknowledge the limitations of our study in the number of cell lines and GSCs used, which might not reflect the molecular heterogeneity and the diversity of *TP53* mutations in GBM. Future studies should aim to include a broader spectrum of GSCs with different *TP53* mutations to further validate our findings.

In this regard, OPK257 exhibited higher sensitivity to Au compared to T98G and similar to that of wtp53-GSCs. This sensitivity can be attributed to the levels of ROS induced by Au and associated with the significant decrease of mutp53 expression in OPK257 (Figure 5a). A ROS-induced decrease of mutp53 expression has been reported in different cancer types. A study investigating the ROS inducer NSC59984 revealed its potential to degrade mutp53 and restore the p53 pathway, an event that was associated with ERK1/2 activation in response to increased ROS [67]. The subcellular localization of phosphorylated ERK can induce cell death by translocating to the nucleus and promoting the activation of pro-apoptotic signaling pathways, such as the intrinsic apoptotic pathway, thereby triggering mitochondrial dysfunction and subsequent apoptotic cell death [68]. Overall, elucidating the intricate connections between TrxR1 activity and p53 function in normal and cancerous cells is imperative, as evidenced by the multifaceted interactions revealed in studies involving p53-targeting compounds such as PRIMA-1, APR-246, MJ25, and RITA, all of which demonstrate distinct yet interconnected pathways involving TrxR1 inhibition and oxidative stress modulation [64].

Remarkably, our results demonstrate the efficacy of co-targeting Trx/GSH systems in GSCs using Au/L-BSO and/or PPL at IC50s within a nanomolar range, regardless of p53 status, underlining its potential as a promising therapeutic strategy in GBM. We demonstrated the synergy between L-BSO and Au in GSCs, which induced significant molecular alterations, such as both AKT dephosphorylation and ERK1/2 phosphorylation, along with the activation of apoptotic pathways and, to some extent, necrotic activation of PARP-1 in all GSCs. Of note, we showed an increase in ERK phosphorylation in GSCs after treatment with Au or Au/LBSO. The localization of p-ERK determines the cell’s fate, and we previously showed the correlation of cytoplasmic p-ERK versus nuclear p-ERK and cell death in GSCs [38]. The necrotic activation of PARP-1 might indicate the presence of extensive DNA damage or deficiencies in DNA repair mechanisms, leading to the sustained activation of PARP-1 [69,70]. The observed lower basal levels of PARP expression in the knockdown p53 GSC line, coupled with the presence of cleaved PARP (89 kDa) indicative of apoptotic cleavage, suggests a potential role played by p53 in apoptotic pathways mediated by PARP. This cleavage has been reported to be predominantly mediated by caspases [71]. Further investigation is needed to fully elucidate the mechanistic link between p53-mediated PARP regulation and the heightened sensitivity to Au observed in the knockdown p53 GSC line.

Using L-BSO to deplete GSH, we provided the proof of principle for the superior efficacy of co-targeting the Trx/GSH systems compared to targeting TrxR alone in GSCs and GBM cell lines. Recognizing the potential challenges posed by the clinical use of L-BSO, in terms of its limitations for safety and crossing the blood-brain barrier [58], we selected PPL, a natural anticancer alkaloid compound capable of crossing the blood-brain barrier and selectively killing cancer cells over normal cells [59]. The natural compound PPL alone exhibited greater potency against GSCs than L-BSO alone. Notably, the knockdown p53 line demonstrated reduced sensitivity to L-BSO, while both the mutp53 and knockdown p53 lines showed diminished sensitivity to PPL. In contrast, the GSC with wtp53 knockdown showed increased sensitivity to Au. This underscores the higher dependence of the GSH system on p53 compared to the Trx system. Notably, PPL demonstrated potency superior to that of L-BSO and exceptional synergy with Au in GSCs. The potential dependency on GSTP1, the primary target of PPL, compared to GCLC might stem from its direct role in neutralizing ROS via glutathione conjugation. The pivotal role of GCLC lies in catalyzing the formation of γ-glutamylcysteine, a precursor for GSH synthesis, dictating its rate and overall production [15]. Remarkably, even at a mere 0.5 µM concentration, PPL significantly reduced Au IC50 to nanomolar range, well below the reported safe concentrations in vivo [59]. These findings suggest that PPL, due to its potent synergistic effect with Au, could be a more effective and selective approach than L-BSO for targeting both GSCs and the tumor bulk GBM cells. Additional studies need to assess the extent of GSH depletion following L-BSO or PPL treatment and the subsequent ROS increase.

## 5. Conclusions

Repurposing the pan-TrxR inhibitor Auranofin, which is FDA-approved for rheumatoid arthritis (Ridaura^®^ La Jolla, CA, USA) has gained increasing interest among cancer researchers and more recently in GBM [35]. Our study provides the first evidence for the role of wtp53-TrxR1axis in regulating response of GSCs to Au and the prospect of implementing a dual-targeting strategy with concomitant synergistic inhibition of Trx/GSH antioxidant systems irrespective of p53 status in GSCs and GBM cell lines in vitro. Dependency on the GSH antioxidant system unravels the importance of targeting it to overcome GSH’s compensation mechanisms to sensitize GBM cell lines and GSCs to Au-induced cell death. The synergistic combination of Au with PPL revealed GSTP1 as a promising therapeutic vulnerability in GSCs. The potent synergy with GCLC and GSTP1 inhibitors in GSCs might provide an efficient and well-tolerated strategy to overcome the resistance of GBM to current therapies. Our study paves the way for preclinical assessment of combined Au and PPL in vivo, heralding a shift to efficiently and safely exploit strategies targeting redox homeostasis for GBM treatment [65].

## Data Availability

The raw data supporting the conclusions of this article will be made available by the authors on request. For data available in publicly accessible repositories, the original data presented in the study are openly available in [The Cancer Genome Atlas (TCGA)] at https://www.cancer.gov/tcga, Cell 2013 database at https://www.cbioportal.org/ and the GlioVis web application at http://gliovis.bioinfo.cnio.es/.

## Supplementary Materials

The following supporting information can be downloaded at: https://www.mdpi.com/article/10.3390/antiox13101201/s1, Table S1. Key resources; Table S2. List of Abbreviations; Figure S1. Graph represents the difference between basal ROS level in OPK49ev and OPK49sh; Figure S2. Western blotting analysis of TrxR1 expression in U87MG with transient transfection of wt-p53; Figure S3. Graphs represent the correlations between Txnrd1 and GSH system members: GSR (a), GSS (b), SGSH (c), OXCT1 (XCT) (d), GPX4 (e), GCLC (f) and GSTP1 (g); Figure S4. The synergy score heatmaps of Au and LBSO combination for T98G (a), U87MG (b), OPK161 (c), OPK257 (d), OPK49ev (e) and OPK49sh (f); Figure S5. Relative densitometric bar graphs of TrxR1, p53, p21, phosphorylated p-AKT (Ser-473), total (T-AKT), phosphorylated ERK1/2 (p-ERK), total ERK1/2 (T-ERK), total (T-PARP-1) and cleaved (C-PARP) apoptotic band (89 kDa). Band intensities were quantified, normalized to actin, and shown relative to the DMSO control. Data represent the mean ± SEM from two independent experiments (* *p* < 0.05 ** *p* < 0.01 *** *p* < 0.001 **** *p* < 0.0001); Figure S6. The synergy score heatmaps of Au and PPL combination for (a) OPK161, (b) OPK257 (c) OPK49.
