# Supplementary material for: Synergistic Dual Targeting of Thioredoxin and Glutathione Systems Irrespective of p53 in Glioblastoma Stem Cells
Source: Antioxidants (Basel). 2024 Oct 3;13(10):1201. doi: 10.3390/antiox13101201 (PMC11504866; doi:10.3390/antiox13101201)

Supplementary Tables

Table S1: Key Resources

REAGENT or RESOURCE	SOURCE	IDENTIFIER
**Antibodies**		
Mouse anti-TrxR1 (B-2), (WB 1:1000)	Santa Cruz Biotechnology Inc.	Cat. No. sc-28321
Mouse anti-GSTP1 (WB 1:10000)	Millipore	Cat. No. ABS1650
Mouse anti-p53 (DO-1), (WB 1:1000)	Santa Cruz Biotechnology Inc.	Cat. No. sc-126
Rabbit anti-p21 Waf1/Cip1 (12D1) (WB 1:1000)	Cell Signaling	Cat. No. 2947
Rabbit anti-Phosphorylated-ERK1/2 (WB 1:1000)	Cell Signaling	Cat. No. 9101S
Rabbit anti-ERK1/2 (WB 1:1000)	Cell Signaling	Cat. No. 9102S
Rabbit anti-p-Akt/Ser473 (193H12) (WB 1:1000)	Cell Signaling	Cat. No. 4058S
Rabbit anti-Akt (WB 1:1000)	Cell Signaling	Cat. No. 9227S
Mouse anti-β-Actin (WB 1:10000)	Cell Signaling	Cat. No. 4967
Rabbit Anti- PARP	Cell Signaling	Cat. No. 9542L
goat anti-mouse IgG (H+L)-HRP conjugate (WB 1:5000)	BioRad Laboratories Inc.	Cat. No. 170-6516
goat anti-rabbit IgG (H+L) conjugate (WB 1:5000)	BioRad Laboratories Inc.	Cat. No. 170-6515
**Chemicals, peptides, and recombinant proteins**		
DMEM	Wisent Inc.	Cat. No. 319-010-CL
DMEM/F12	Wisent Inc.	Cat. No. 319-075-CL
Penicillin-Streptomycin 1:100	Wisent Inc.	450-201-EL
Glutamax 1:100	Wisent Inc.	609-066EL
W21 1:50	Wisent Inc.	003-015-XL
Fetal Bovine Serum (FBS)	Wisent Inc.	080-150
Epidermal Growth Factor (EGF) 10ug/ml	Wisent Inc.	511-110 EU
Fibroblast Growth Factor (FGF)10ug/ml	Wisent Inc.	511-126-EU
Heparin 0.2%	Wisent Inc.	521-020-UL
DMSO	Fisher Scientific	Cat. BP231-100
EDTA 0.53MM	Wisent Inc.	Cat. 32-060-EL
Trypan Blue Solution	Gibco	Lot# 1621894
Crystal violet solution	Sigma-Aldrich	Lot# 061M1821V
Temozolomide	TOCRIS Bioscience	Batch No: 2A/204232
Auranofin	Cayman Chemical Company	Item. 15316
L-BSO	Sigma-Aldrich	Lot# SLCC9195
PiperLongumine	EMD Millipore Corp., USA	Lot# 4059345
**Critical commercial assays**		
BCA Protein Assay Kit	ThermoFisher Scientific	Cat. 23225
ROS Assay	Invitrogen	Lot# 2456929
**Experimental models: Cell lines**		
Human: T98G	American Tissue Culture Collection (ATCC)	
Human: U87MG	American Tissue Culture Collection (ATCC)	HTB-14
Human: OPK161 patient-derived neurosphere cells		N/A
Human: OPK257 patient-derived neurosphere cells		N/A
Human: OPK49 patient-derived neurosphere cells		N/A
Human: OPK49sh		N/A
**Recombinant DNA**		
PLKO.1 shp53 vector	Addgene	No.19119
**Software and algorithms**		
Image Lab Software	BioRad Laboratories Inc.	RRID:SCR_014210
GraphPad Prism 8	GraphPad Software	RRID:SCR_002798
ImageJ	NIH	RRID:SCR_003070
SynergyFinder+	S Zheng · 2022	https://synergyfinder.org/
cBioProtal	Cerami et al., 2012; Gao et al., 2013	https://www.cbioportal.org/
Gliovise	L. Bowman et al., 2017	http://gliovis.bioinfo.cnio.es/

Supplementary Tables

Table S2: List of Abbreviations

ABBREVIATION	MEANING
Au	Auranofin
DMSO	Dimethyl sulfoxide
GBM	Glioblastoma
GCLC	Glutamate-cysteine ligase catalytic subunit
GCS	Glutamylcysteine synthetase
GPX	Glutathione peroxidase
GSC	Glioblastoma stem cell
GSH	Glutathione
GST	Glutathione transferase
GSTP-1	Glutathione S-transferase pi-1
L-BSO	L-buthionine sulfoximine
NAC	N-acetylcysteine
PPL	Piperlongumine
ROS	Reactive oxygen species
RSEM	RNA-Seq by Expectation Maximization
RT	Radiation therapy
SC	Synergy score
TCGA	The Cancer Genome Atlas
TMZ	Temozolomide
Trx	Thioredoxin
TrxR	Thioredoxin reductase
WT	Wild-type

Figure S1.

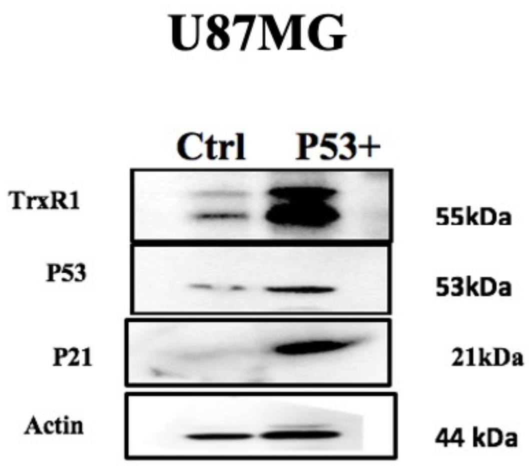

Figure S2.

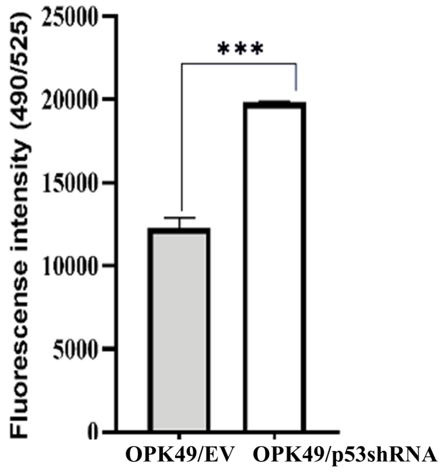

Figure S3.

a.

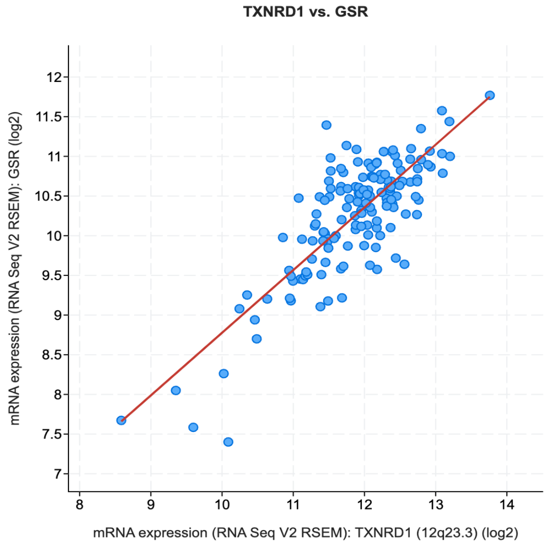

Pearson: 0.80(p = 7.84e-36)

b.

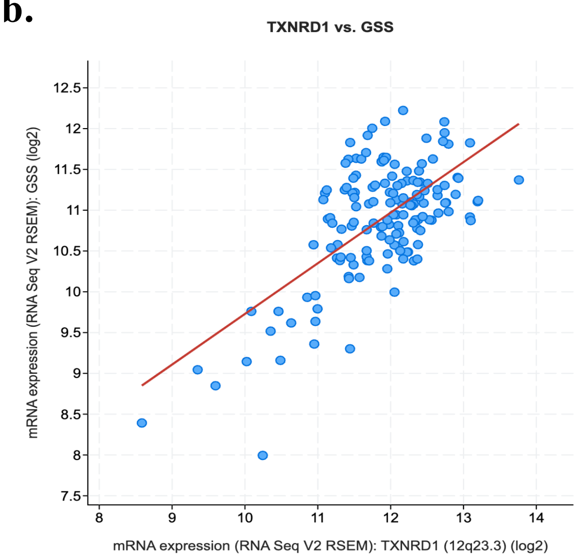

Pearson: 0.65(p = 1.68e-19)

c.

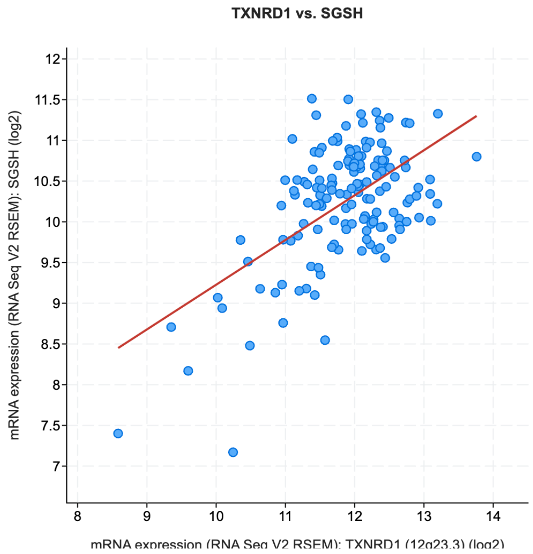

Pearson: 0.57(p = 3.09e-14)

d.

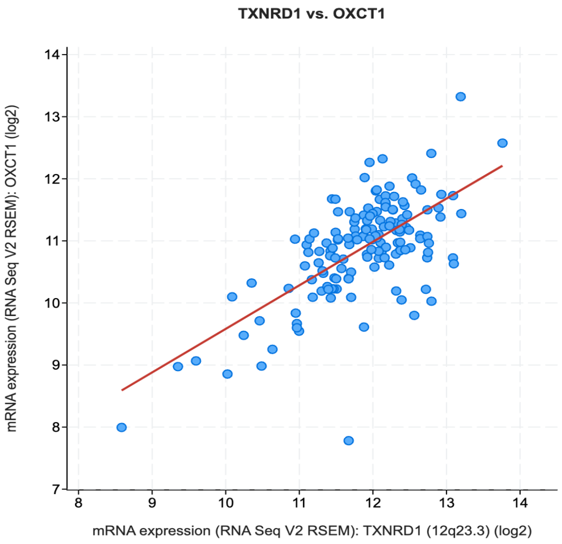

Pearson: 0.66(p = 2.70e-20)

e.

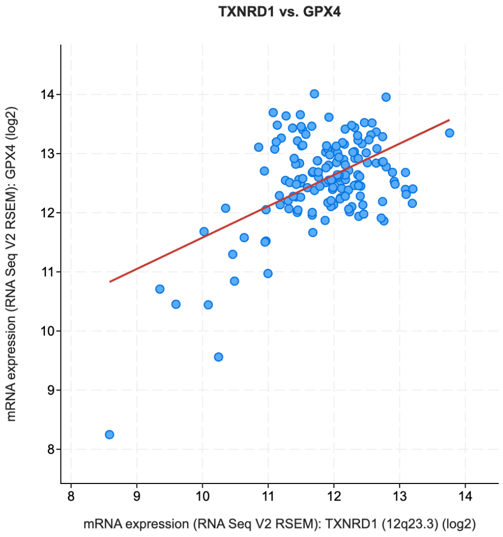

Pearson: 0.52(p = 4.64e-12)

f.

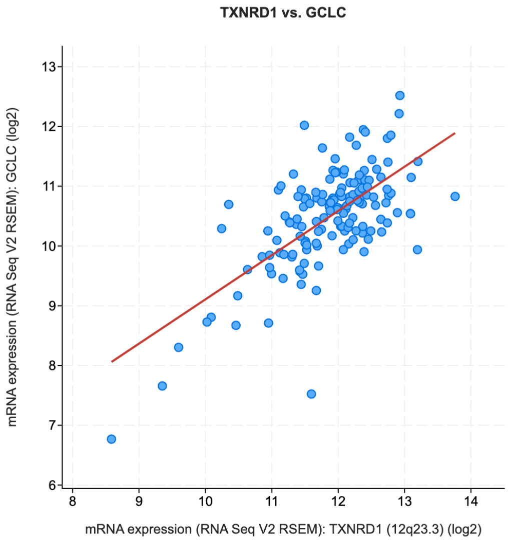

Pearson: 0.70(p = 5.46e-21)

g.

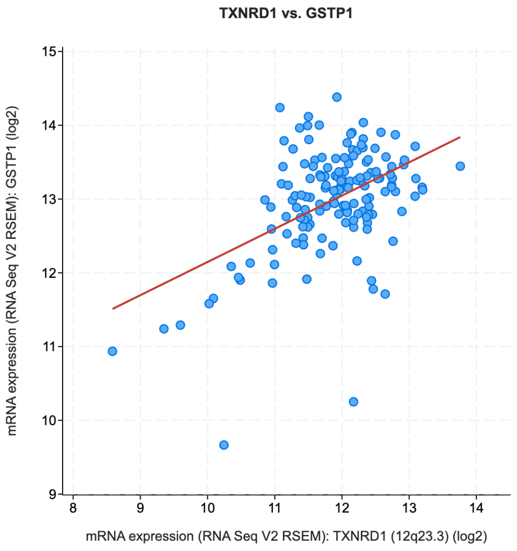

Pearson: 0.50(p = 3.58e-10)

Figure S4. Synergy Score Heatmaps of Au and L-BSO Combination

a. T98G: Au+(1 and 5 uM L-BSO)

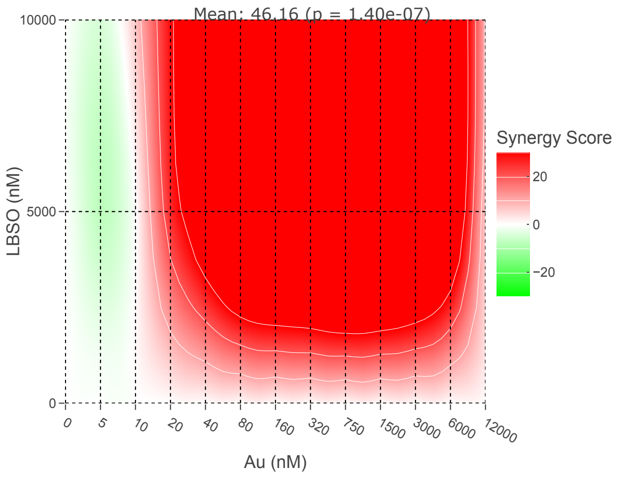

b. U87MG : Au+(1 and 5 uM L-BSO)

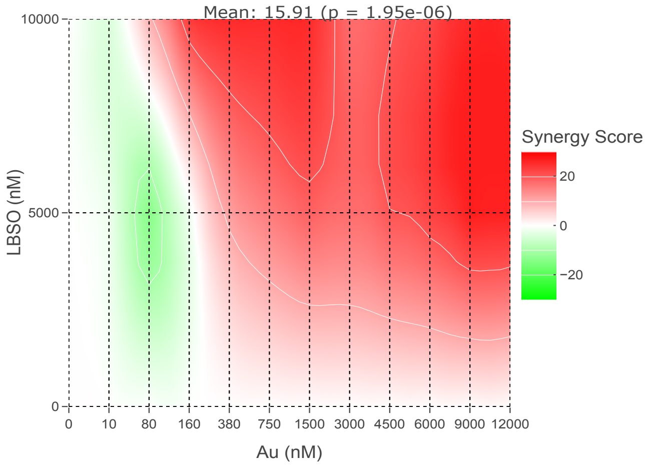

c. OPK161: Au+(1, 5, 10 uM L-BSO)

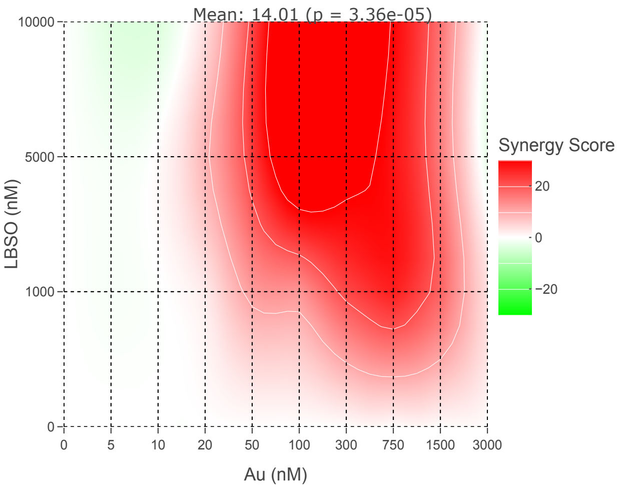

d. OPK257: Au+(1, 5, 10 uM L-BSO)

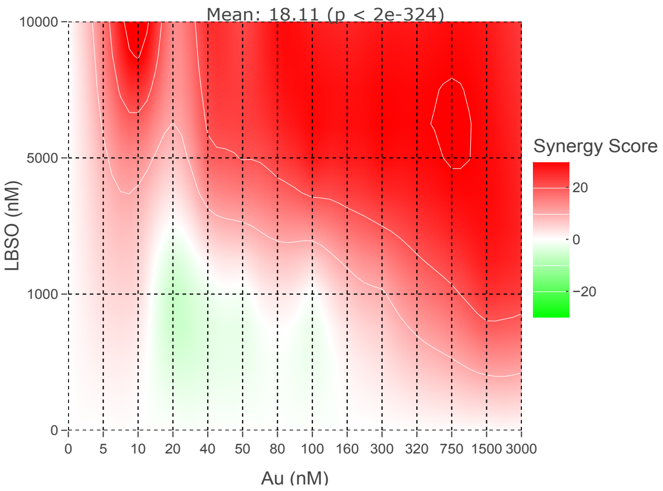

e. OPK49ev: Au+(1, 5, 10 uM L-BSO)

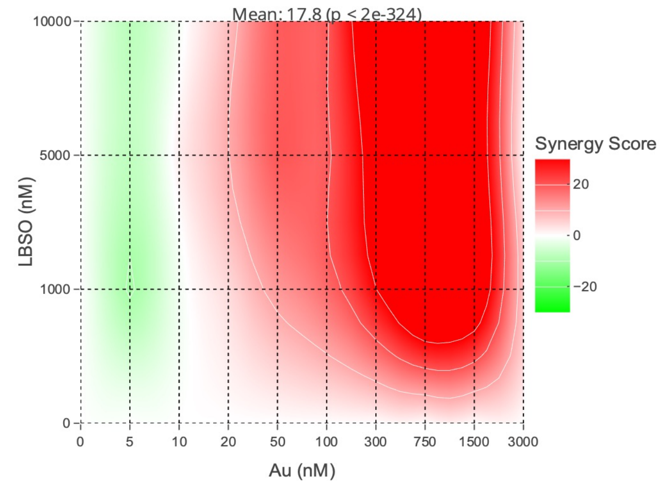

f. OPK49sh: Au+(1, 5, 10 uM L-BSO)

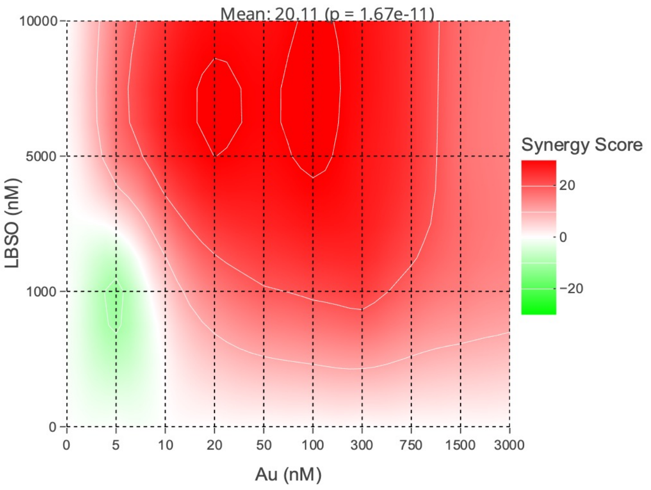

Figure S5. Representative Quantification of Western Blots

a.

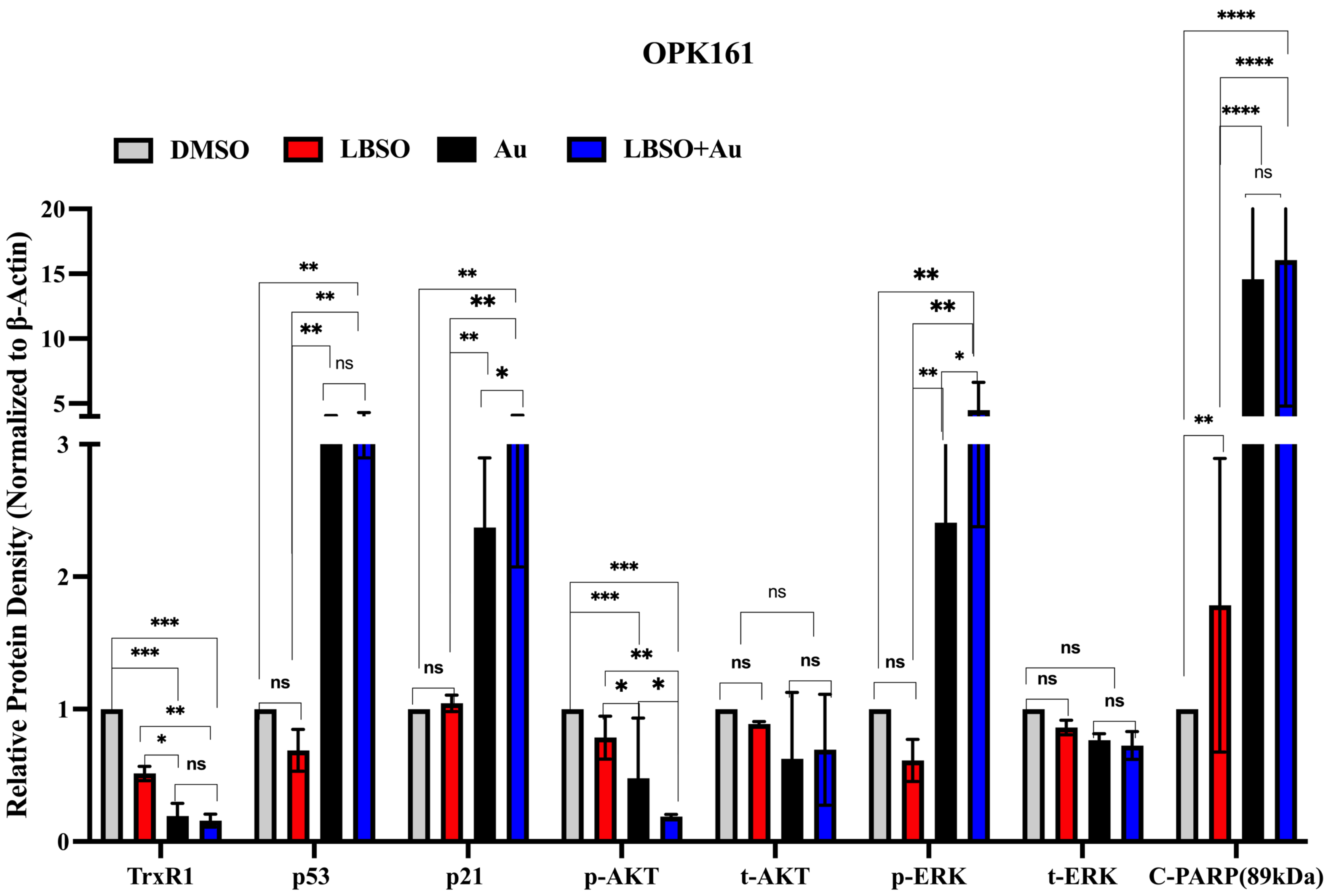

b.

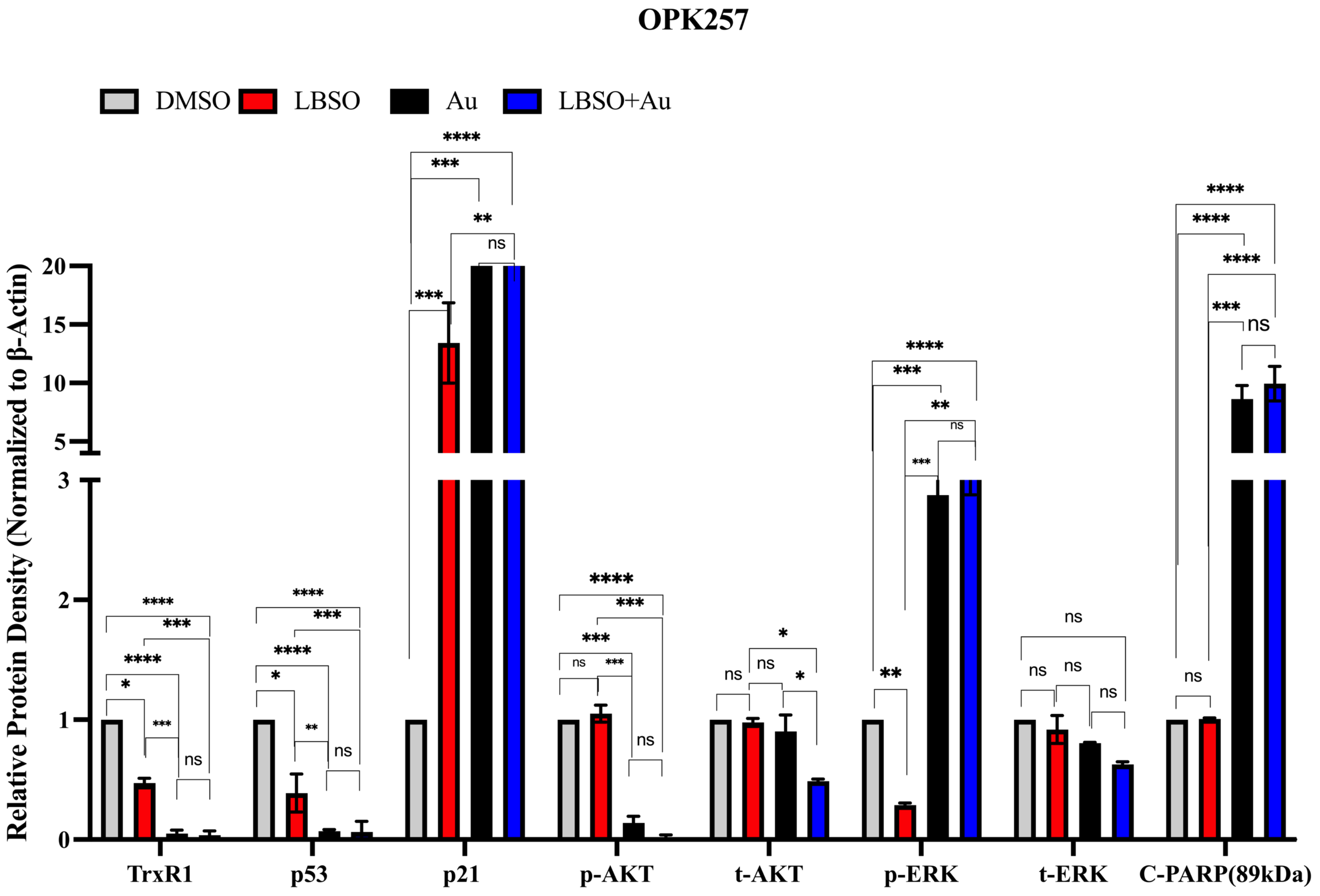

c.

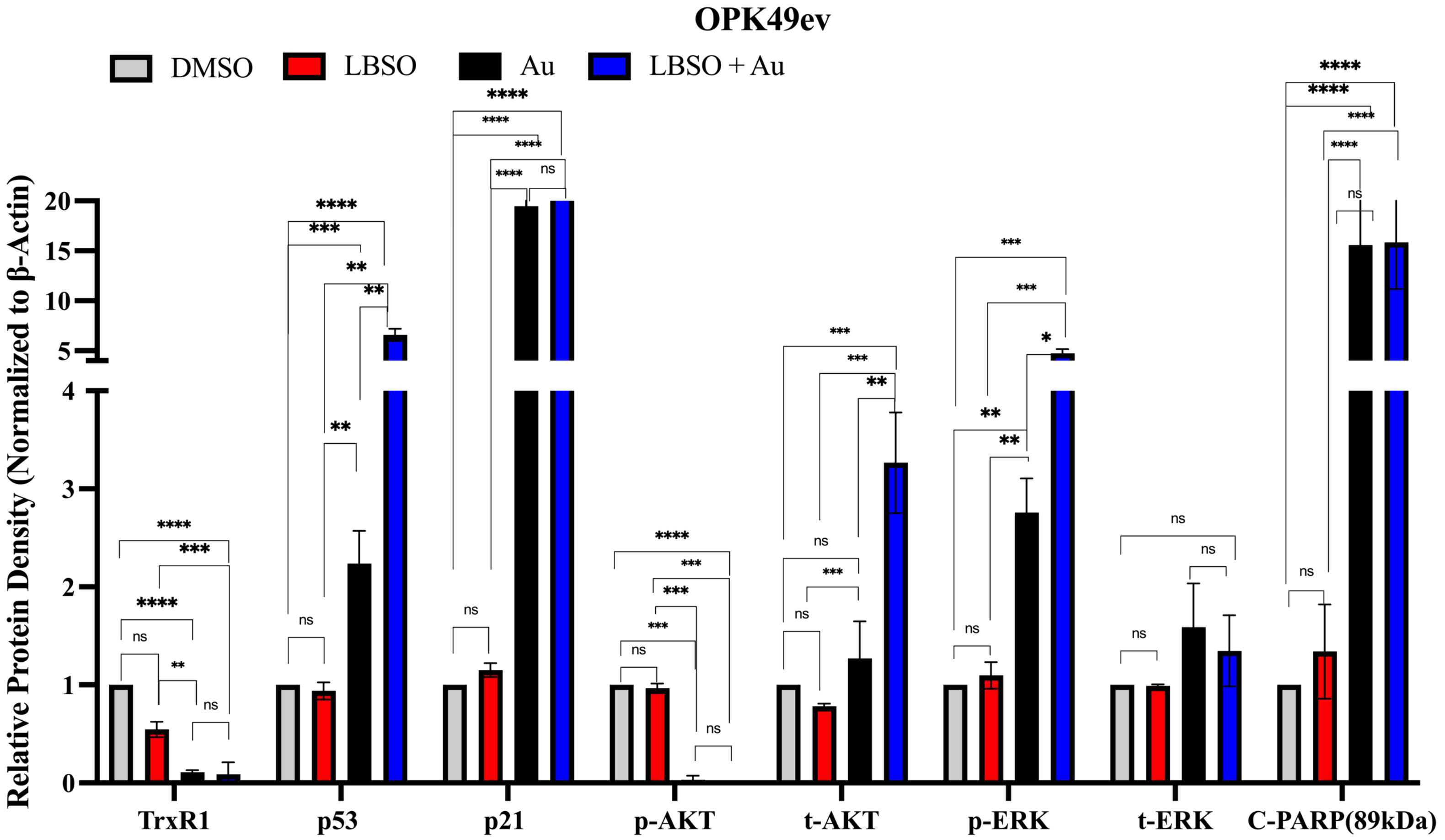
OPK49ev
DMSO
LBSO
Au
LBSO + Au
Relative Protein Density (Normalized to β-Actin)
TrxR1
p53
p21
p-AKT
t-AKT
p-ERK
t-ERK
C-PARP(89kDa)

d.

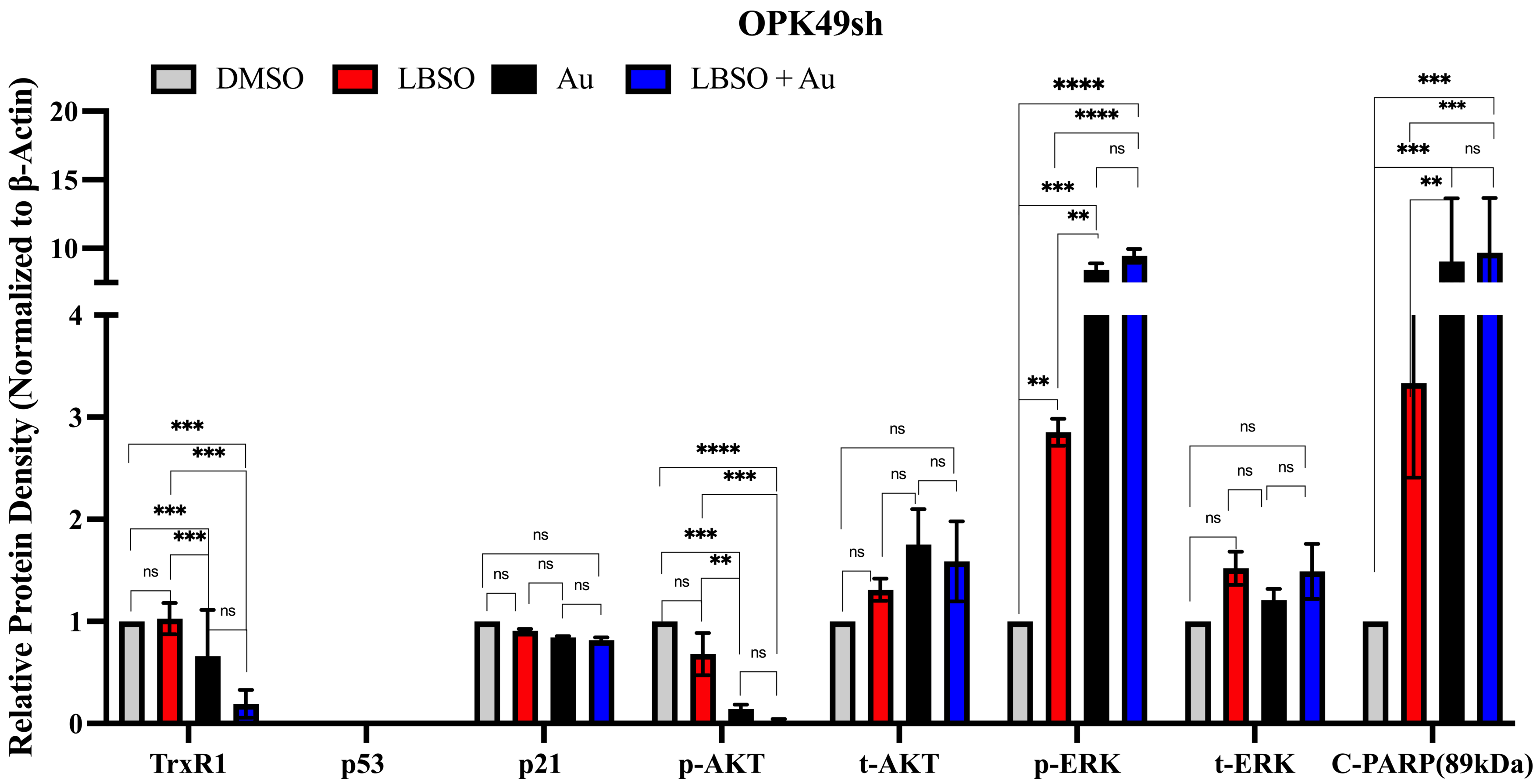
OPK49sh
DMSO
LBSO
Au
LBSO + Au
Relative Protein Density (Normalized to β-Actin)
TrxR1
p53
p21
p-AKT
t-AKT
p-ERK
t-ERK
C-PARP(89kDa)

Figure S6.

a. OPK161: Au+(0.5 uM PPL)

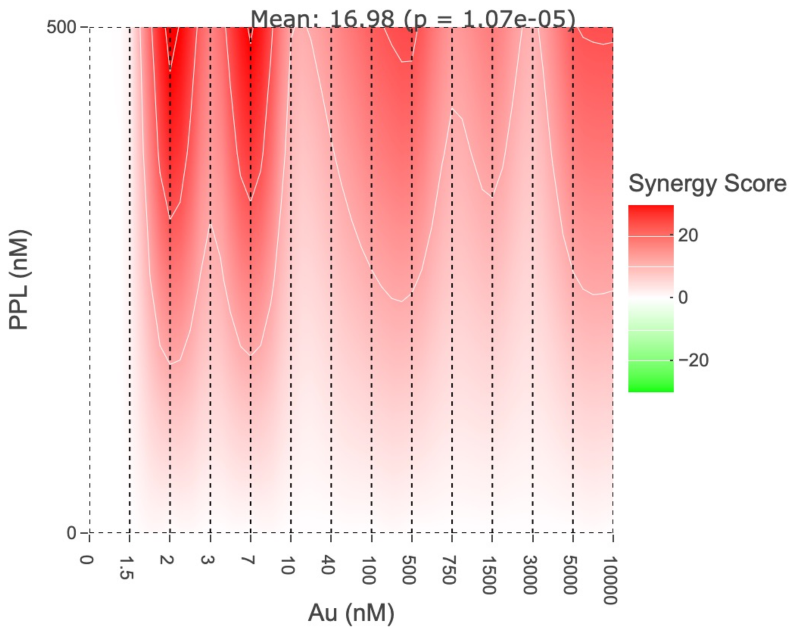

b. OPK49: Au+(0.5 uM PPL)

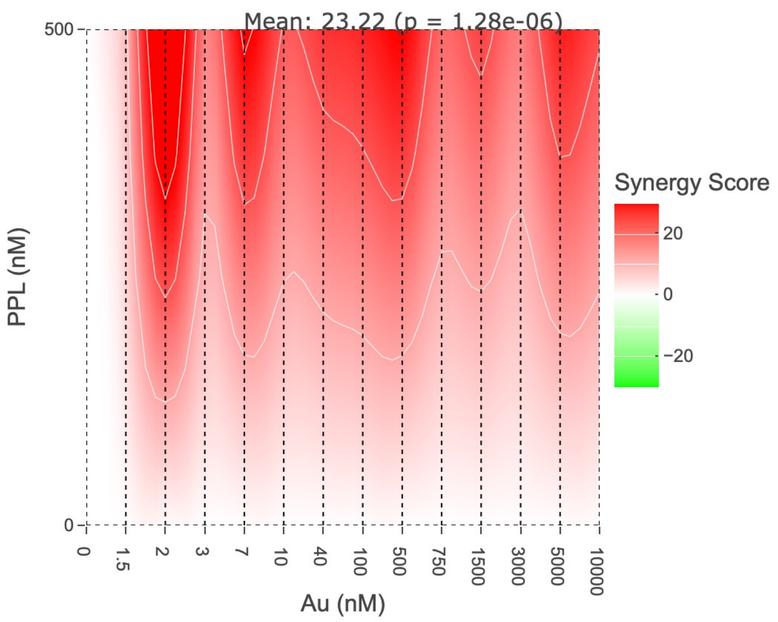

c. OPK257: Au+(0.5 uM PPL)

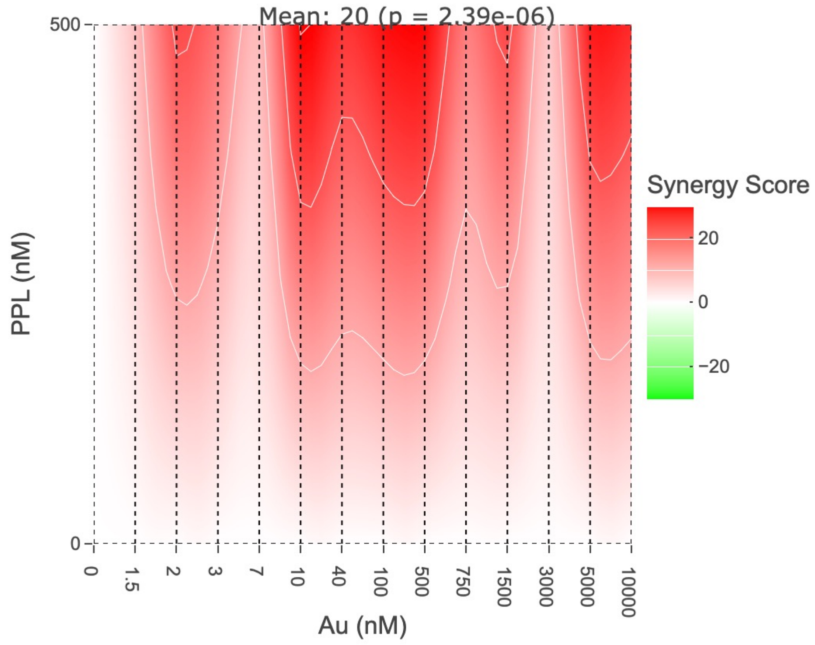

Supplement: Supplementary file 1 [file antioxidants-13-01201-s001.zip › antioxidants-3180313-supplementary.pdf]
